# Synthesis and Study of the Ru-Pt Heterometallic Complexes [RuCp(L) (PPh_3_)-µ-dmoPTA-1κ*P*:2κ^2^-*N,N′*-Pt(κ^2^C*,O*-CH_2_N(CH_3_)CHO)][PtCl_4_] (L = Cl, PPh_3_)

**DOI:** 10.1155/bca/4247392

**Published:** 2025-10-28

**Authors:** Andrés Alguacil, Franco Scalambra, Adrián Puerta, Aday González-Bakker, José M. Padrón, Antonio Romerosa

**Affiliations:** ^1^Inorganic Chemistry Section-CIESOL, University of Almería, Almería, Spain; ^2^BioLab, Instituto Universitario de Bio-Orgánica Antonio González, Universidad de la Laguna, C/ Astrofísico Francisco Sánchez 2, 38206 La Laguna, Spain

**Keywords:** antiproliferative activity, heterometallic complexes, platinum, ruthenium

## Abstract

Complexes [RuCp(PPh_3_)_2_(HdmoPTA)][PtCl_4_] (**1**), [RuCp(PPh_3_)_2_-µ-dmoPTA-1κ*P*:2κ^2^-*N,N′*-Pt(κ^2^*C,O*-CH_2_N(CH_3_)CHO)][PtCl_4_] (**2**), [RuClCp(PPh_3_) (HdmoPTA)]_2_[PtCl_4_] (**3**), and [RuClCp(PPh_3_)-µ-dmoPTA-1κ*P*:2κ^2^-*N,N′*-Pt(κ^2^*C,O*-CH_2_N(CH_3_)CHO)]_2_[PtCl_4_] (**4**) have been synthesized and characterized by NMR, IR, and crystal structure of **2** was obtained by single crystal X-Ray diffraction. Antiproliferative activity of **1** was assessed against six human solid tumor cell lines and compared to cisplatin as a standard, showing GI_50_ values in the submicromolar range.

## 1. Introduction

The research of anticancer compounds based on coordination complexes is something on which much effort and resources have been devoted for decades [1–3], especially since the discovery of the antiproliferative activity of cisplatin [4]. The promising results shown by this platinum-based drug resulted in extensive studies on its mechanism of action and in the development of many derivatives that, together with cisplatin, some of them are still in use today [5–8]. However, despite the high activity shown by platinum compounds, they also exhibit several adverse side effects that have hindered their further development [9–12].

For this reason, research efforts were focused on searching for coordination compounds based on other transition metals [13–17]. Of the metals studied, the most promising is ruthenium due to its wide range of oxidation states and coordination geometries, as well as its ability to biomimic iron by interacting with bloodstream proteins [18–20]. Numerous ruthenium compounds have been synthesized for this purpose, some of them entering advanced stages of clinical trials (KP1019, KP1339, NAMI-A) [21] and being evaluated in preclinical trials such as RAPTA-C [22, 23]. The interesting antitumor profile of the complexes belonging to the RAPTA family has led to the synthesis of numerous derivatives, of which we recently reported the substitution of the PTA ligand (1,3,5-triaza-7-phosphaadamantane) [24] by a dmoPTA ligand (3,7-dimethyl-1,3,7-triaza-5-phosphabicyclo[3.3.1]nonane) [25] in its structure, giving rise to the [Ru(η^6^−C_10_H_14_) (Cl_2_) (dmoPTA)] complex from which the respective heterodimetallic complexes [Ru(η^6^−C_10_H_14_) (Cl_2_)-μ-dmoPTA-1κ*P*:2κ^2^*N,N′*-MCl_2_] (M = Zn, Co, Ni) could be obtained [26].

Due to the synergistic effect produced by the presence of two transition metals, the synthesis of heterodimetallic compounds has been a topic in which our group has put a lot of effort, especially since we reported the synthesis of the Ru-Zn compound [RuCp(PPh_3_)_2_-µ-dmoPTA-1κ*P*:2κ^2^*N,N′*-ZnCl_2_](CF_3_SO_3_) [27], which showed anticancer activities up to 200 times higher than those shown by cisplatin against several cancer cell lines. Since that point, different Ru-M (M = Zn, Co, Cu, Ni, Pd) polyheterometallic complexes have been synthesized [28], but so far the Ru-Pt combination had not been contemplated, and the already good anticancer activity results of the precursor monometallic complex [RuCp(PPh_3_)_2_(HdmoPTA)](CF_3_SO_3_)_2_ together with the evidences shown by different Ru-Pt complexes found in the literature led us to investigate the possible antiproliferative properties of the Ru-Pt analog. With this aim, this work presents the reactivity of [RuCp(PPh_3_)_2_(HdmoPTA)](CF_3_SO_3_)_2_ and [RuClCp(PPh_3_) (HdmoPTA)](CF_3_SO_3_) with K_2_[PtCl_4_]. The obtained compounds were characterized, and the antiproliferative activity of **1** was tested against a panel of cancer cells.

## 2. Results and Discussion

### 2.1. Synthesis and Characterization of 1–4

**Scheme 1:**
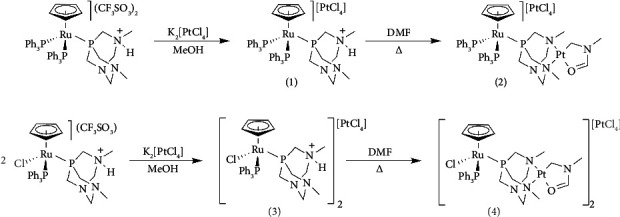
Synthesis of **1**–**4**.

Reacting one equivalent of K_2_[PtCl_4_] with [RuCp(PPh_3_)_2_(HdmoPTA)](CF_3_SO_3_)_2_ in MeOH, complex [RuCp(PPh_3_)_2_(HdmoPTA)][PtCl_4_] (**1**) precipitated as a deep yellow powder after 30-min stirring. When dissolved in DMF at 27°C, after 24 h crystals of [RuCp(PPh_3_)_2_-µ-dmoPTA-1κ*P*:2κ^2^-*N,N′*-Pt(κ^2^C,O-CH_2_N(CH_3_)CHO)][PtCl_4_] (**2**) (Scheme 1) were obtained. A similar strategy was used for the synthesis of [RuClCp(PPh_3_) (HdmoPTA)]_2_[PtCl_4_] (**3**) from the reaction of K_2_[PtCl_4_] with [RuClCp(PPh_3_) (HdmoPTA)](CF_3_SO_3_) in MeOH and subsequent dissolution of **3** in DMF at 37°C for 24 h to obtain [RuClCp(PPh_3_)-µ-dmoPTA-1κ*P*:2κ^2^-*N,N′*-Pt(κ^2^*C,O*-CH_2_N(CH_3_)CHO)]_2_[PtCl_4_] (**4**) (Scheme 1).

The ^31^P{^1^H} NMR spectra of **1** and **3** show the distinctive resonances of the PPh_3_ and HdmoPTA ligands. For **1**, a doublet at 38.81 ppm and a triplet at −15.19 ppm in a 2:1 ratio are observed in DMSO-d_6_, corresponding to PPh_3_ and HdmoPTA, respectively (Figures S2 and S18). The ^1^H NMR (Figure S1) of **1** aggress with the proposed composition shown in Scheme 1. In the case of **3**, signals ascribable to PPh_3_, and HdmoPTA appears at 46.26 ppm and −4.15 ppm, respectively, as two doublets (^2^*J*_PP_ = 43.8, 43.5 Hz). The counterions of both Complexes **1** and **3** were corroborated by means of ^195^Pt{^1^H} NMR, where [PtCl_4_]^2−^ appears at −1428 ppm in DMF-d_7_ and -2954 ppm in DMSO-d_6_, which are the expected chemical shift for [PtCl_4_]^2−^ and [PtCl_3_(DMSO-κ*S*)] ^−^, respectively [29] The infrared spectra of **1** and **3** are very similar to those of the complex [RuCp(PPh_3_)_2_(HdmoPTA)](CF_3_SO_3_)_2_, except that for the distinctive strong stretching band of the triflate anion is absent (Figures S15 and S23, respectively). However, for Complexes **2** and **4** (Figures S16 and S24, respectively), the characteristic CO stretching absorption of the DMF ligand is observed at 1665 and 1666 cm^−1^, respectively, shifted almost 10 cm^−1^ to lower frequencies with respect to pure DMF.

For Complexes **2** and **4**, NMR characterization was not possible due to the lack of solubility, so their composition was confirmed by elemental analysis and by single crystal X-ray diffraction for **2**.

### 2.2. Crystal Structure of 2

**Figure 1:**
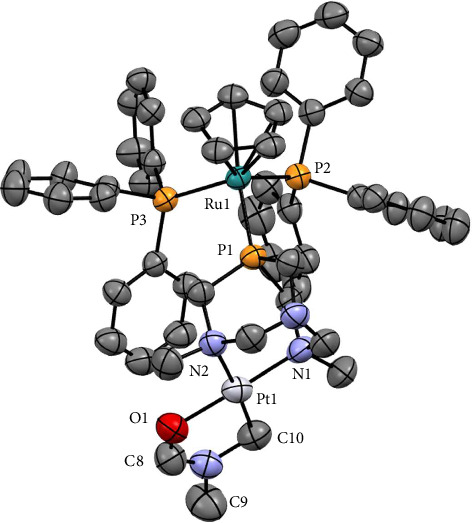
Thermal ellipsoid representation (50% probability) of one complex cation in the crystal structure of **2**. Complex anion and hydrogen atoms have been omitted for the sake of clarity.

Complex **2** crystallized in yellow needles by slow evaporation of solutions of **1** in DMF at 37°C. The asymmetric unit contains two dimetallic moieties [RuCp(PPh_3_)_2_-µ-dmoPTA-1κ*P*:2κ^2^-*N,N′*-Pt(κ^2^*C,O*-CH_2_N(CH_3_)CHO)]^2+^ (Figure 1) and two [PtCl_4_]^2−^ counterions. Selected bond lengths and angles are given in Table 1, while complete lists can be found in Tables S3-S4.

The coordination sphere of the ruthenium atom in **2** displays a piano-stool geometry, bearing a Cp two PPh_3_ and dmoPTA ligand, which bonds the ruthenium through its P atom and a [Pt(κ^2^*C,O*-CH_2_N(CH_3_)CHO)]^+^ unit *via* the methylated nitrogen. The Ru-Cp_centroid_ distance (1.876 Å) is in the same range of similar ruthenium half sandwich complexes (from 1.836 to 1.893 Å) [30]. The ruthenium–phosphorus bond lengths are slightly shorter (Ru1-P1 = 2.310(5) Å) than in the parent complex [RuCp(PPh_3_)_2_(HdmoPTA)]^2+^ (Ru-P_dmoPTA_ = 2.389(1) Å) [30].

The distance between the N_CH3_ atoms (d_N1-N2_ = 2.91(3) Å) is significantly shorter with respect to the deprotonated monometallic complex [RuCp(PPh_3_)_2_(dmoPTA)]^+^ (3.609(4) Å) [31] but slightly longer than in the protonated form of the same complex, [RuCp(PPh_3_)_2_(HdmoPTA)]^2+^ (2.800(6) Å) [30]. On the other hand, the N1-N2 distance is also somewhat shorter than in the dimetallic complexes [RuCp(PPh_3_)_2_-µ-dmoPTA-1κ*P*:2κ^2^-*N,N′*-ZnCl_2_](CF_3_SO_3_) and [RuCp(PPh_3_)_2_-µ-dmoPTA-1κ*P*:2κ^2^-*N,N′*-CoCl_2_](CF_3_SO_3_) (RuZn: 2.935-2.962(6) Å; RuCo: 2.941-2.953(5) Å) [28, 30].

The most surprising feature of the structure of **2** is that platinum completes its coordination sphere with a C-metalated (κ^2^C,O-CH_2_N(CH_3_)CHO) moiety that originates from DMF and is formed through its sp^3^ C-H activation. Dimethylformamide is a well-known source of C-, N-, or CN-based functional groups *via* the more common sp^2^ or the less common sp^3^ C-H activation. At the best of our knowledge, only few examples of crystal structures featuring a M-C(sp^3^) bonded DMF are reported and contains Ir(III), Pt(II), or Pt(IV) [32, 33]. The Pt-C bond length found in **2** (Pt1-C10 = 2.07(1) Å) is slightly more similar to the Ir-C distance found for the reported Ir(III) complex than the Pt-C distances in the Pt(II) and Pt(IV) complexes, being 2.065(1) Å for Ir(III)-C, 2.019(6) Å - 2.031(5) Å for Pt(II)-C and 2.018(4) Å–2.042(4) Å for Pt(IV)-C.

### 2.3. Antiproliferative Activity Against Human Solid Tumor Cell Lines

The initial assessment of the antiproliferative activity of Complex **1** was carried out in a panel of six human solid tumor cell lines. The results, expressed as growth inhibition 50% (GI_50_) after 48 h exposure, are shown in Table 2. The standard anticancer drug cisplatin was selected as reference drug for comparison purposes. Complex [RuCp(PPh_3_)_2_(HdmoPTA)][PtCl_4_] (**1**) was able to induce cell growth inhibition at the submicromolar range in all cell lines.

The GI_50_ values are in the range of 0.08–2.2 μM for Complex **1**. The results are not as promising as in the case of tetranuclear complexes [{RuCp(PPh_3_)_2_-µ-dmoPTA-1κ*P*:2κ^2^-*N,N′*-CuCl}_2_-µ-Cl-µ-OCH_3_](CF_3_SO_3_)_2_·(CH_3_OH)_4_ and [{RuCp(PPh_3_)_2_-µ-dmoPTA-1κ*P*:2κ^2^-*N,N′*-NiCl}_2_-µ-Cl-µ-OH](CF_3_SO_3_)_2_ [34], but at the same level as potent [RuCp(PPh_3_)_2_-µ-dmoPTA-1κ*P*:2κ^2^-*N,N′*-ZnCl_2_](CF_3_SO_3_) precursor complex, showing remarkable antiproliferative activity against all cell lines tested [27]. The cell line where it shows the highest anticancer activity is the cervical cancer cell line HeLa, where it exhibits values in the nanomolar concentration range which, despite being preliminary tests, are even higher than those shown by the most effective compounds against this cell line [35, 36]. Stability tests of **1** in DMSO-d_6_ and DMSO-d_6_/cell medium 1:1 were conducted by ^31^P{^1^H} NMR on samples incubated at 37°C (Figures S25 and S26). For what concern the DMSO-d_6_ solution, after 30 min, a pair of doublets at δ(^31^P) = −5.89 ppm and δ(^31^P) = −43.22 ppm were observed, together with O=PPh_3_, that are consistent with the formation of [RuCp(DMSO-κ*S*) (HdmoPTA) (PPh_3_)](CF_3_SO_3_)_2_ [34]. Over time, the amount of this species slightly increases, and after 24 h also another pair of doublets starts to form at δ(^31^P) = −3.42 ppm and δ(^31^P) = 46.89 ppm that can be assessed to [RuClCp(HdmoPTA) (PPh_3_)](CF_3_SO_3_). After 48 h, the amount of Complex **1** in the solution was still the 91%. On the other hand, in the tests run with DMSO-d_6_/cell medium 1:1, the peaks corresponding to PPh_3_ and HdmoPTA appear as broad bands with maxima at δ(^31^P) = 40.20 ppm and δ(^31^P) = −11.97, respectively. After 4 h, a shoulder starts to appear next to the band of PPh_3_, displaced by 2.3 ppm downfield. This shoulder reached a ratio of approximately 1:9 with respect to PPh_3_ after 48 h. No evidence of free PPh_3_ was observed.

## 3. Conclusions

Complexes [RuCp(PPh_3_)_2_(HdmoPTA)][PtCl_4_] (**1**), [RuCp(PPh_3_)_2_-µ-dmoPTA-1κ*P*:2κ^2^-*N,N′*-Pt(κ^2^*C,O*-DMF)][PtCl_4_] (**2**), [RuClCp(PPh_3_) (HdmoPTA)]_2_[PtCl_4_] (**3**), and [RuClCp(PPh_3_)-µ-dmoPTA-1κ*P*:2κ^2^-*N,N′*-Pt(-κ^2^*C,O*-DMF)]_2_[PtCl_4_] (**4**) have been synthesized. Complexes **1** and **3** have been characterized by multinuclear NMR, IR, and elemental analysis, while Complex **2** has been characterized by IR and single-crystal X ray diffraction. Antiproliferative activity of Complex **1** against six human solid tumor cell lines was determined displaying submicromolar values of GI_50_ against all tested cell lines, reaching nanomolar values of GI_50_ in Hela cervix cancer cell line, result that improves the antiproliferative activity of previous similar complexes synthesized against this cell line and that showed by similar complexes found in the literature.

## Figures and Tables

**Table 1:** Selected bond lengths and angles of **2**.

Bond lengths (Å)		2	Bond angles (°)			2
Pt1	N2	2.150 (8)	O1	Pt1	N2	93.5 (3)
Pt1	O1	2.032 (7)	O1	Pt1	N1	177.3 (3)
Pt1	N1	2.040 (9)	O1	Pt1	C10	82.6 (4)
Pt1	C10	2.066 (11)	N1	Pt1	N2	87.3 (3)
Ru1	P3	2.375 (2)	N1	Pt1	C10	96.6 (4)
Ru1	P1	2.309 (2)	C10	Pt1	N2	175.8 (4)
Ru1	P2	2.391 (2)	Cl3	Pt2	Cl1	179.11 (14)
Ru1	C48	2.220 (9)	Cl1	Pt2	Cl2	91.26 (15)
Ru1	C47	2.210 (9)	P3	Ru1	P2	100.79 (7)
Ru1	C50	2.228 (8)	P1	Ru1	P3	94.74 (8)
Ru1	C49	2.243 (8)	P1	Ru1	P2	98.21 (8)
Ru1	C51	2.219 (8)				

**Table 2:** GI_50_ values (μM) of cisplatin and **1** against human solid tumor cell lines.

	Cell lines					
	A549 (lung)	HBL-100 (breast)	HeLa (cervix)	SW1573 (lung)	T-47D (breast)	WiDr (colon)
Cisplatin	4.9 ± 0.2	1.9 ± 0.2	1.8 ± 0.5	2.7 ± 0.4	17 ± 3.3	23 ± 4.3
[RuClCp(PPh_3_)(HdmoPTA)]^+^	—	—	2.6 ± 0.2	1.5 ± 0.1	1.9 ± 0.5	1.7 ± 0.4
**1**	1.3 ± 0.12	0.93 ± 0.12	0.08 ± 0.014	0.34 ± 0.040	2.2 ± 0.29	1.4 ± 0.20

*Note:* [RuClCp(PPh_3_)(HdmoPTA)]^+^ and cisplatin GI_50_ values taken, respectively, from ref 16 and 20.

## Data Availability

The data that support the findings of this study are available in the supporting information of this article.

## Supporting Information

Electronic supporting information (ESI) available: Experimental procedures, ^1^H, ^31^P and ^13^C, ^1^H-^1^H COSY, ^1^H-^13^C HSQC, ^1^H-^15^N HMBC, ^195^PT NMR spectra, IR spectra, X-ray diffraction analysis of CCDC 2401376, stability in DMSO and DMSO/aqueous, IC_50_ values.
