# Supplementary material for: Synthesis and Study of the Ru-Pt Heterometallic Complexes [RuCp(L) (PPh3)-µ-dmoPTA-1κP:2κ2-N,N′-Pt(κ2C,O-CH2N(CH3)CHO)][PtCl4] (L = Cl, PPh3)
Source: Bioinorg Chem Appl. 2025 Oct 28;2025:4247392. doi: 10.1155/bca/4247392 (PMC12585859; doi:10.1155/bca/4247392)
Supplement: Supporting Information — Additional supporting information can be found online in the Supporting Information section. [file 4247392.f1.docx]

## *Supporting information*

Synthesis and study of the Ru-Pt heterometallic complexes [RuCp(L)(PPh_3_)-µ-dmoPTA-1κ*P*:2κ^2^-*N,N’*-Pt(κ^2^*C,O*-CH_2_N(CH_3_)CHO)][PtCl_4_] (L = Cl, PPh_3_).

Andrés Alguacil,^a^ Franco Scalambra,^a^ Adrián Puerta,^b^ Aday González-Bakker, ^b^ José M. Padrón,^b^ Antonio Romerosa ^a*^

*^a^ Área de Química Inorgánica-CIESOL, Universidad de Almería, Almería, Spain*

*^b^ BioLab, Instituto Universitario de Bio-Orgánica “Antonio Gónzalez”, Universidad de La Laguna, C/ Astrofísico Francisco Sánchez 2, 38071 La Laguna, Spain*

**Keywords:** Ruthenium, platinum, heterodimetallic complexes, antiproliferative activity

**Contents**

General Procedures3

Synthesis of [RuCp(HdmoPTA)(PPh_3_)_2_][PtCl_4_] (**1**)3

Synthesis of [RuCp(PPh_3_)_2_-µ-dmoPTA-1ĸ*P*:2ĸ^2^*N,N’*-Pt(DMF- ĸ^2^*C,O*)][PtCl_4_] (**2**)20

Synthesis of [RuClCp(HdmoPTA)(PPh_3_)]_2_[PtCl_4_] (**3**)21

Synthesis of [RuClCp(PPh_3_)-µ-dmoPTA-1ĸ*P*:2ĸ^2^*N,N’*-Pt(DMF-ĸ^2^*C,O*)]_2_[PtCl_4_] (**4**)30

Stability tests31

Compound cytotoxicity evaluated using the MTT assay33

Single crystal X-ray diffraction data34

References36

**General Procedures**

All chemicals were reagent grade and, unless otherwise stated, were used as received by commercial suppliers. The solvents were all degassed and distilled according to standard procedures. All reactions and manipulations were routinely performed under a dry nitrogen atmosphere by using standard Schlenk-tube techniques. The hydrosoluble phosphine PTA,^1^ dmPTA(CF_3_SO_3_)_2_,^2^ [RuClCp(PPh_3_)_2_],^3^ [RuClCp(PPh_3_)(HdmoPTA)](CF_3_SO_3_),^2^ [RuCp(PPh_3_)_2_(HdmoPTA)](CF_3_SO_3_)_2_,^4^ were prepared as described in the literature. NMR spectra were recorded with a Bruker Avance III Hd 500 MHz NMR instrument operating at 500.13 MHz (^1^H), 125.76 MHz (^13^C), 202.46 MHz (^31^P), 50.68 MHz (^15^N) and 107.51 MHz (^195^Pt) equipped with a BBFO probe or with a Bruker Avance III HD 600 MHz NMR instrument operating at 600.13 MHz (^1^H), 150.92 MHz (^13^C), 242.94 MHz (^31^P), 60.81 MHz (^15^N) equipped with a QCI-P CryoProbe^TM^. Peak positions are relative to tetramethylsilane and were calibrated against the residual solvent resonance (^1^H) or the deuterated solvent multiplet (^13^C). Chemical shifts for ^31^P{^1^H} NMR were measured relative to external 85% H_3_PO_4_ with downfield values taken as positive. For ^15^N and ^195^Pt NMR chemical shifts were measured relative to external CH_3_NO_2_ and K_2_[PtCl_4_], respectively. Elemental analysis (C, H, N, S) were performed on a Fisons Instruments EA 1108 elemental analyser. The solubility of the synthesized complexes was determined by UV-vis spectrophotometry techniques.

### **Synthesis of [RuCp(HdmoPTA)(PPh_3_)_2_][PtCl_4_] (1)**

A solution of K_2_[PtCl_4_] (24.5 mg, 0.059 mmol) in H_2_O (200 µL) was added dropwise into a solution of [RuCp(PPh_3_)_2_(HdmoPTA-ĸ*P*)](CF_3_SO_3_)_2_ (68.6 mg, 0.059 mmol) in dry MeOH (10 mL). After 30 minutes of stirring, a yellow-coloured precipitate is obtained. This yellow precipitate was suction filtered, washed with Et_2_O (3 x 10 mL) and dried under vacuum. Yield: 40.9 mg, 57%, S_25ºC,DMSO_ 3.4 mg/cm^3^, S_25ºC,DMF_ 2.8 mg/cm^3^. C_48_H_52_Cl_4_N_3_P_3_PtRu (PM = 1201.8 g·mol^-1^). Calc: C 47.99; H 4.37; N 3.50. Found: C 47.78; H 4.15; N 3.19. ^1^H NMR (600.13 MHz, DMSO-d_6_, 25ºC) δ (ppm): 2.30 (6H, bs, N*CH_3_*), 3.40 + 3.42 (1H + 1H, m + m, P*CH_2_*N), 3.44 + 3.70 (2H + 2H, m + d, ^2^*J*_HH_ = 11.77 Hz, P*CH_2_*NCH_3_), 3.88 + 4.21 (2H + 2H, d + d, ^2^*J*_HH_ = 11.27 Hz, ^2^*J*_HH_ = 11.09 Hz, N*CH_2_*N), 5.07 (5H, s, Cp), 6.89 + 7.47 + 7.56 (30H, m + m + m, aromatics, PPh_3_). ^13^C{^1^H} NMR (150.90 MHz, DMSO-d_6_, 25ºC) δ (ppm): 41.82 + 41.96 + 42.10 + 42.24 (s + s + s + s, (*CH_3_*)_2_SO), 42.94 (d, ^3^*J*_PC_ = 4.65 Hz, N*CH_3_*), 48.54 (s, P*CH_2_*N), 55.50 + 55.61 (s + s, P*CH_2_*NCH_3_), 73.42 (N*CH_2_*N), 85.76 (Cp), 129.05-136.41 (PPh_3_). ^15^N NMR (60.81 MHz, DMSO-d_6_, 25ºC) δ (ppm): 47.28 (PCH_2_*N*), 45.73 (NCH_2_*N*). ^31^P{^1^H} NMR (242.94 MHz, DMSO-d_6_, 25ºC) δ (ppm): 38.81 (d, ^2^*J*_pp_ = 39,55Hz, PPh_3_), -15.19 (dd, ^2^*J*_PP_ = 39.21Hz, ^1^*J*_PC_ = 38.74Hz, dmoPTA). ^15^N NMR (60.81 MHz, DMF-d_7_, 25ºC) δ (ppm): 45.73 (*N*CH_2_*N*), 47.28 (PCH_2_*N*). ^195^Pt{^1^H} NMR (64.32 MHz, DMSO-d_6_, 25ºC) δ (ppm): -2954.38 (s, [PtCl_3_(DMSO-κ*S*)]^-^). ^1^H NMR (600.13 MHz, DMF-d_7_, 25ºC) δ (ppm): 2.67 (6H, bs, N*CH_3_*), 3.40 + 3.54 (1H + 1H, m + m, P*CH_2_*N), 3.66 + 4.44 (2H + 2H, d + d, ^2^*J*_HH_ = 13.47 Hz, ^2^*J*_HH_ = 14.13 Hz, P*CH_2_*N_CH3_), 4.17 + 4.51 (2H + 2H, d + d, ^2^*J*_HH_ = 11.55 Hz, ^2^*J*_HH_ = 11.77 Hz, N*CH_2_*N), 5.25 (5H, s, Cp), 7.04 + 7.58 + 7.62 (30H, m + m + m, aromatics, PPh_3_). ^13^C{^1^H} NMR (150.90 MHz, DMF-d_7_, 25ºC) δ (ppm): 43.52 (d, ^3^*J*_PC_ = 4.64 Hz, N*CH_3_*), 49.46 (s, P*CH_2_*N), 57.32 + 57.44 (m, P*CH_2_*N_CH3_), 74.65 (d, ^3^*J*_PC_ = 3.31 Hz, N*CH_2_*N), 86.64 (s, Cp), 129.73-137.41 (PPh_3_). ^31^P{^1^H} NMR (242.94 MHz, DMF-d_7_, 25ºC) δ (ppm): 38.63 (d, ^2^*J*pp = 37.85Hz, PPh_3_), -13.18 (dd, ^2^*J*_PP_ = 39.28Hz, ^2^*J*_PC_ = 39.59Hz, dmoPTA). ^15^N NMR (60.81 MHz, DMF-d_7_, 25ºC) δ (ppm): 46.64 (*N*CH_2_*N*), 47.69 (PCH_2_*N*). ^195^Pt{^1^H} NMR (107.51 MHz, DMF-d_7_, 25ºC) δ (ppm): -1428.72 (bs, PtCl_4_). IR (ATR, cm^-1^): 606(w), 621(w), 641(w), 648(w), 664(w), 682(w), 699(s), 732(w), 746(m), 760(m), 806(w), 821(w), 832(w), 852(w), 861(w), 930(w), 998(w), 1021(w), 1072(w), 1091(m), 1106(w), 1144(w), 1182(w), 1225(w), 1279(w), 1323(w), 1415(w), 1435(m), 1479(w), 2836(w), 2880(w), 2908(w), 3070(w).

**
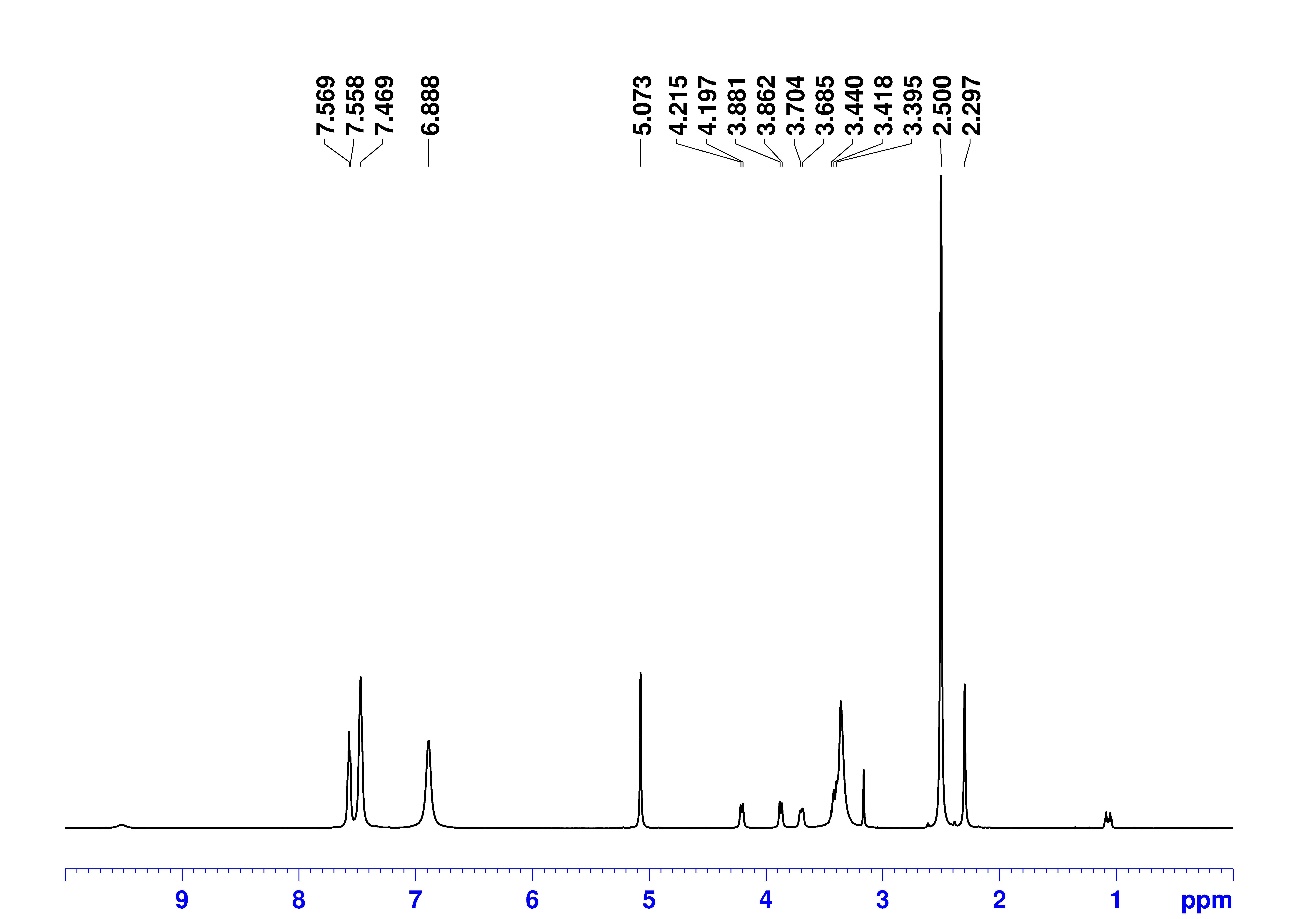
**

**Figure S1**. ^1^H NMR (600.13MHz, DMSO-d_6_, 25ºC) of **1**

**
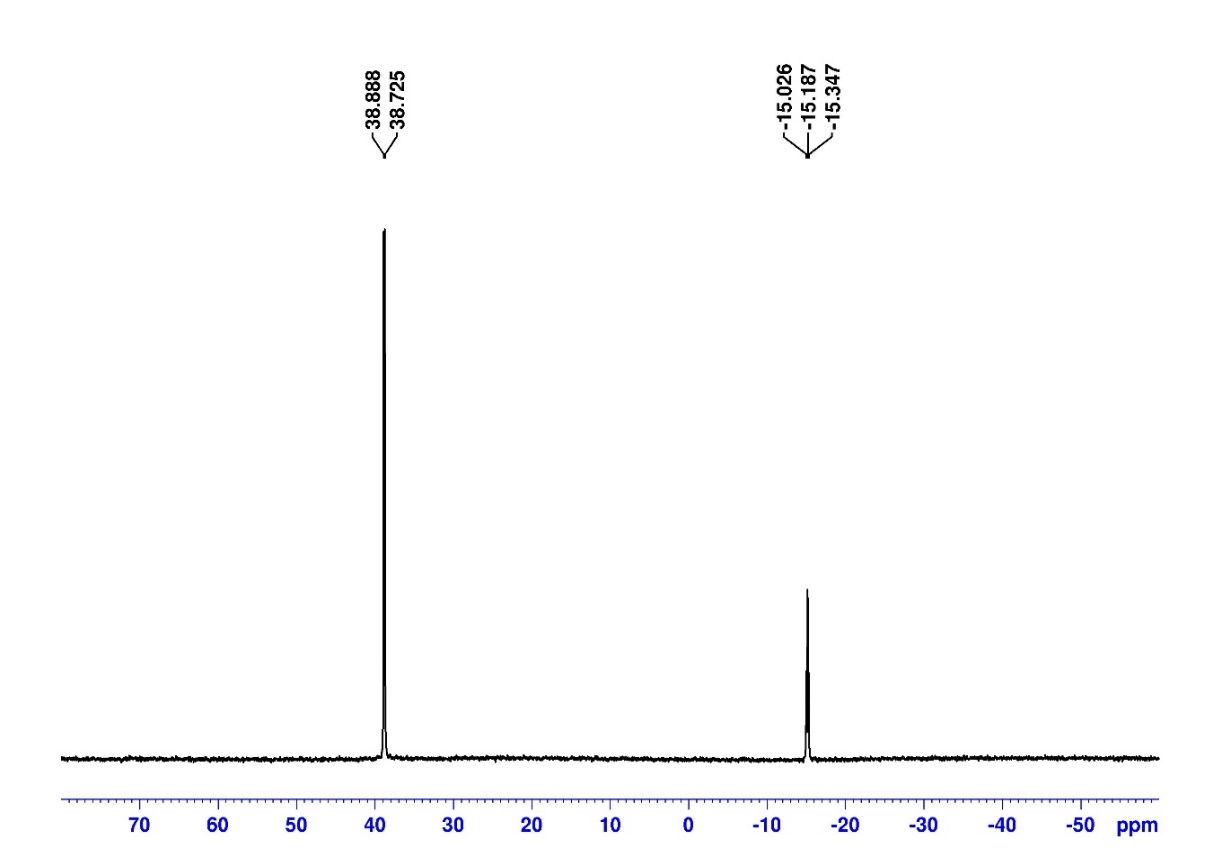
**

**Figure S2**. ^31^P{^1^H} NMR (242.94MHz, DMSO-d_6_, 25ºC) of **1**

**
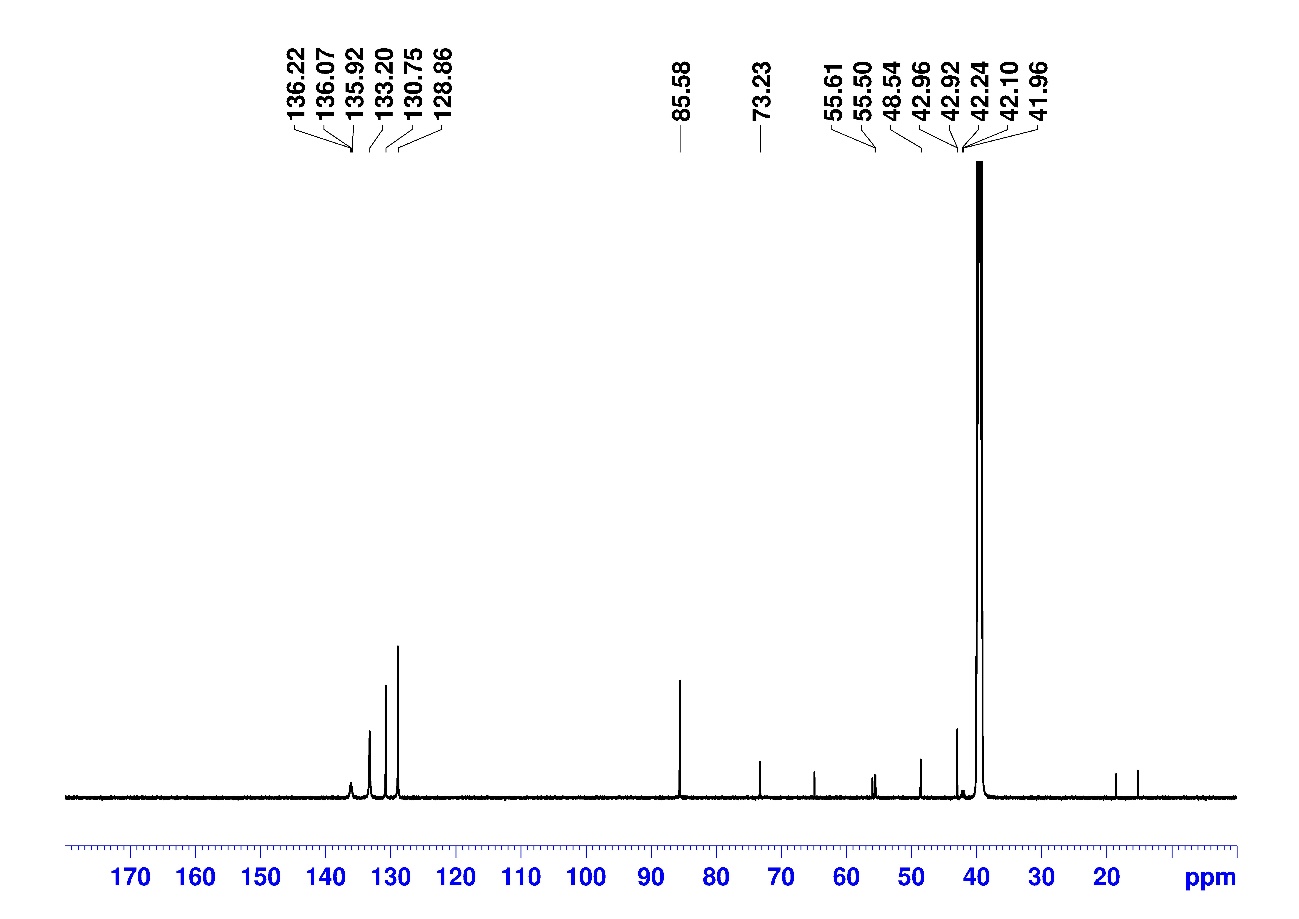
**

**Figure S3**. ^13^C{^1^H} NMR (150.90MHz, DMSO-d_6_, 25ºC) of **1**

**
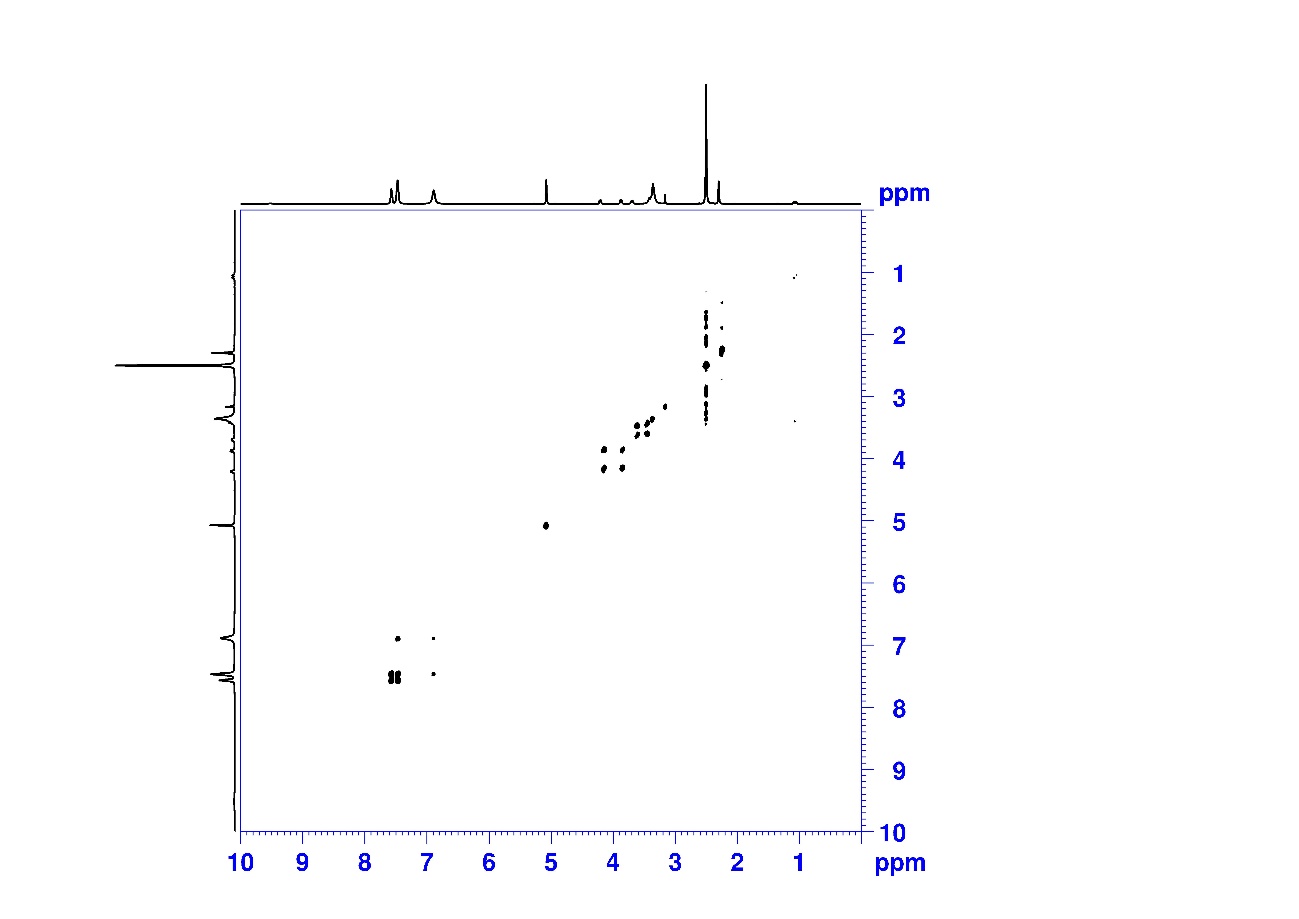
**

**Figure S4**. ^1^H-^1^H COSY NMR (600.13MHz, 600.13MHz, DMSO-d_6_, 25ºC) of **1**


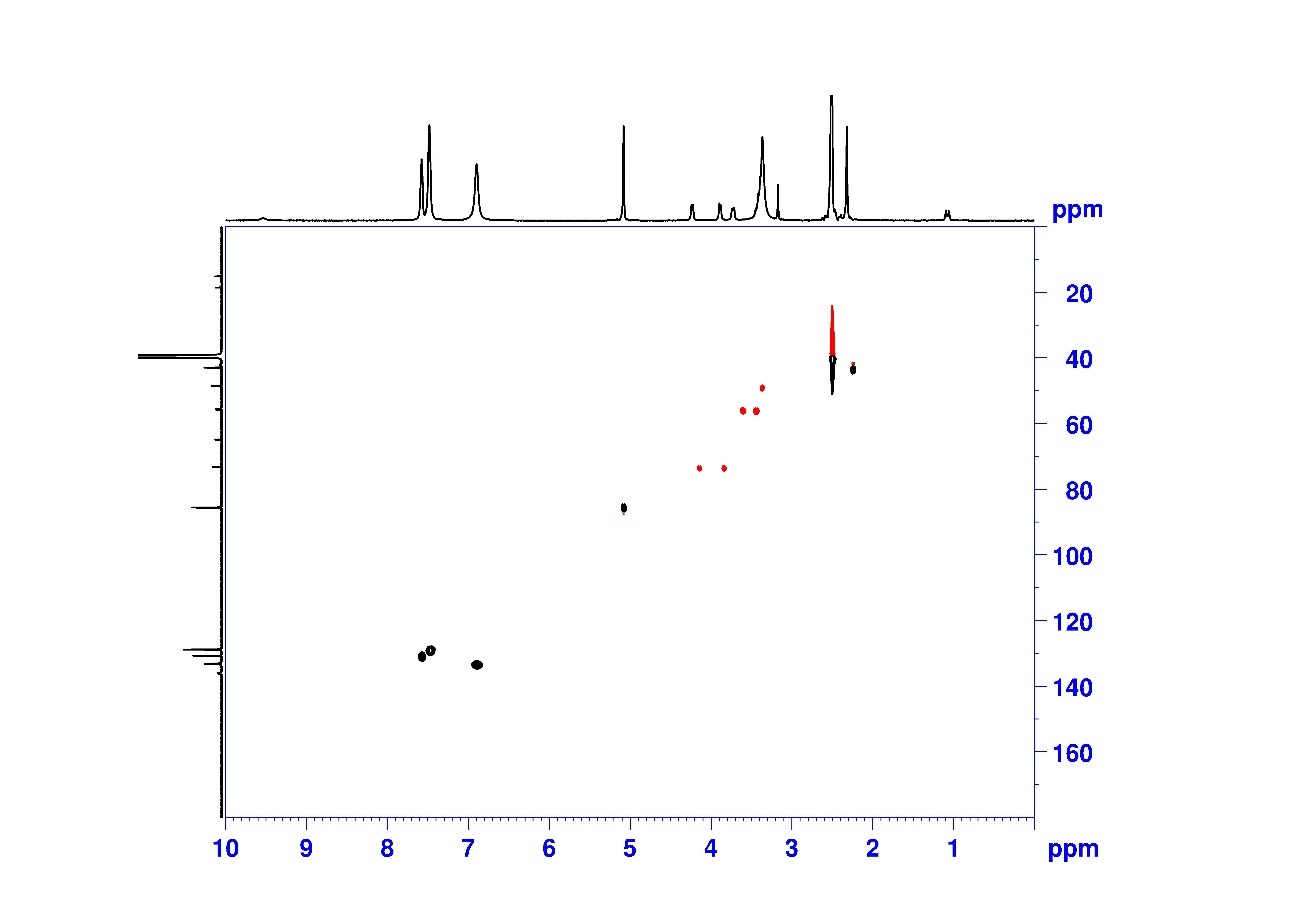


**Figure S5**. ^1^H-^13^C HSQC NMR (600.13MHz, 150.90MHz, DMSO-d_6_, 25ºC) of **1**.

**
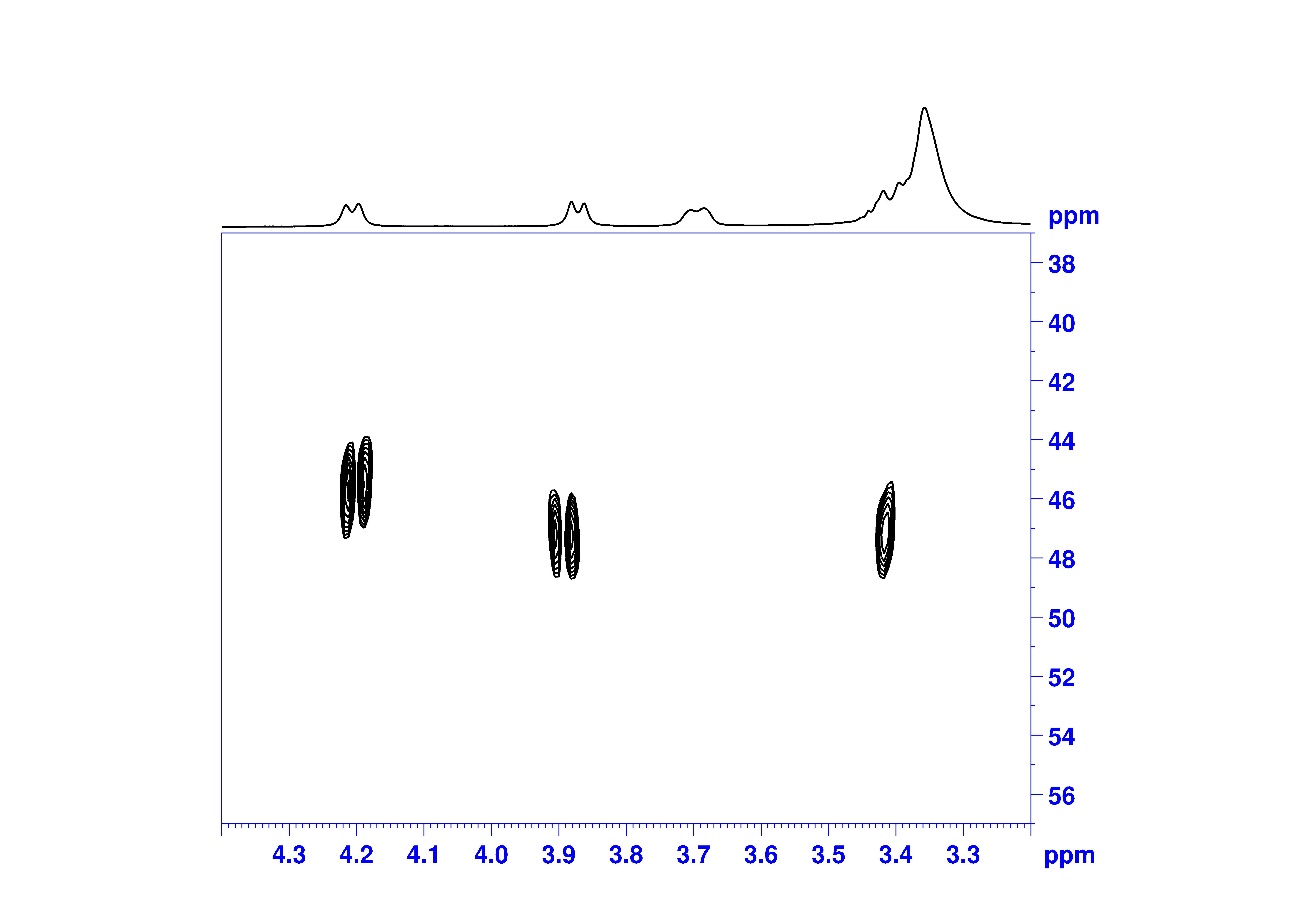
**

**Figure S6**. ^1^H-^15^N HMBC NMR (600.13MHz, 60.81MHz, DMSO-d_6_, 25ºC) of **1**.

**
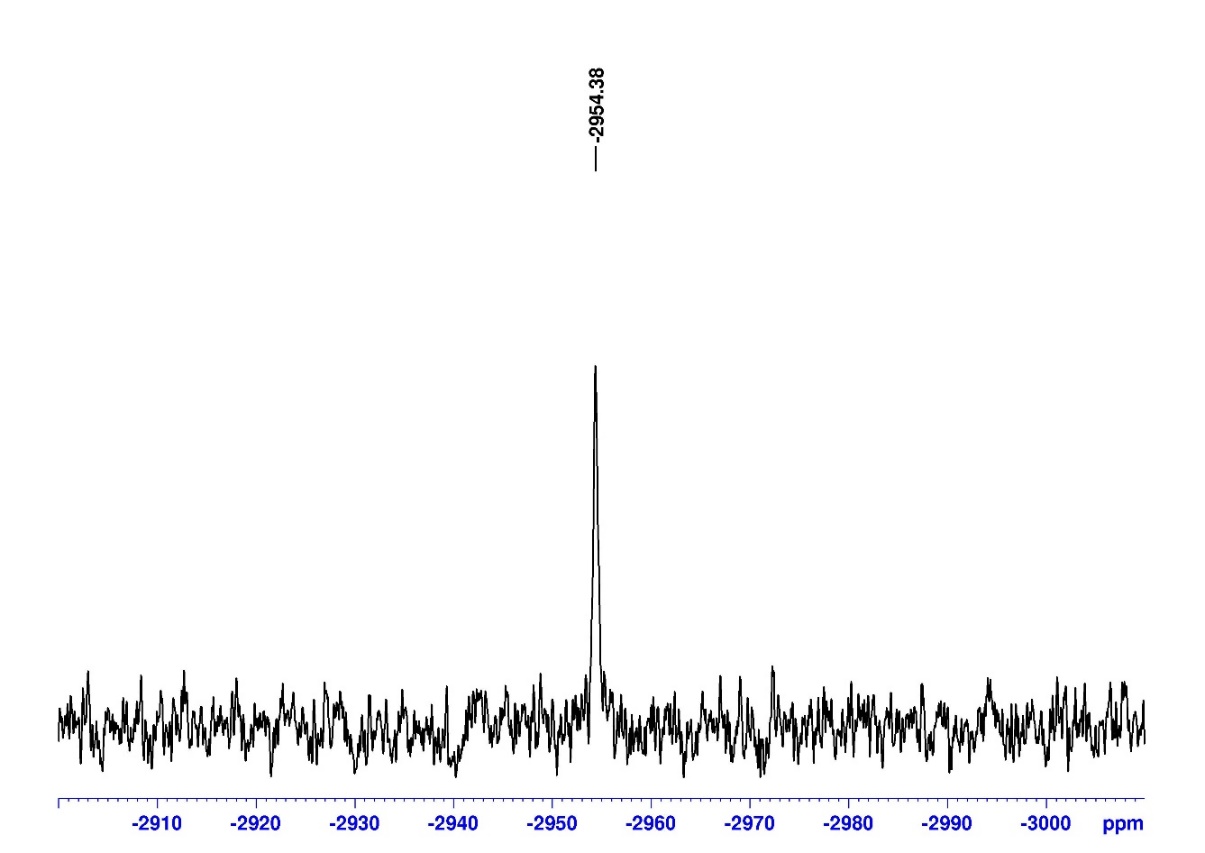
**

**Figure S7**. ^195^Pt{^1^H} NMR (64.51 MHz, DMSO-d_6_, 25ºC) of **1**

**
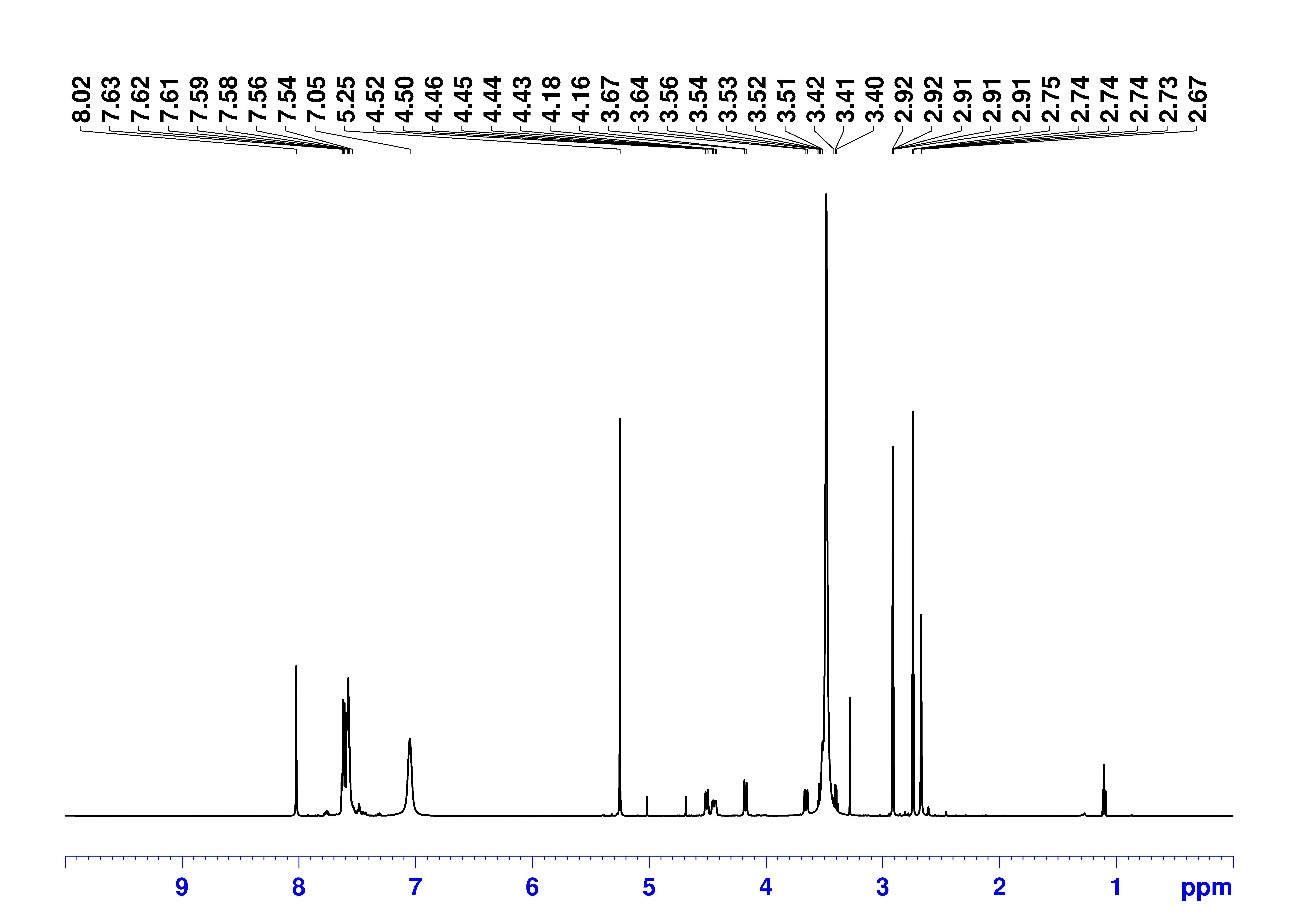
**

**Figure S8**. ^1^H NMR (600.13MHz, DMF-d_7_, 25ºC) of **1**

**
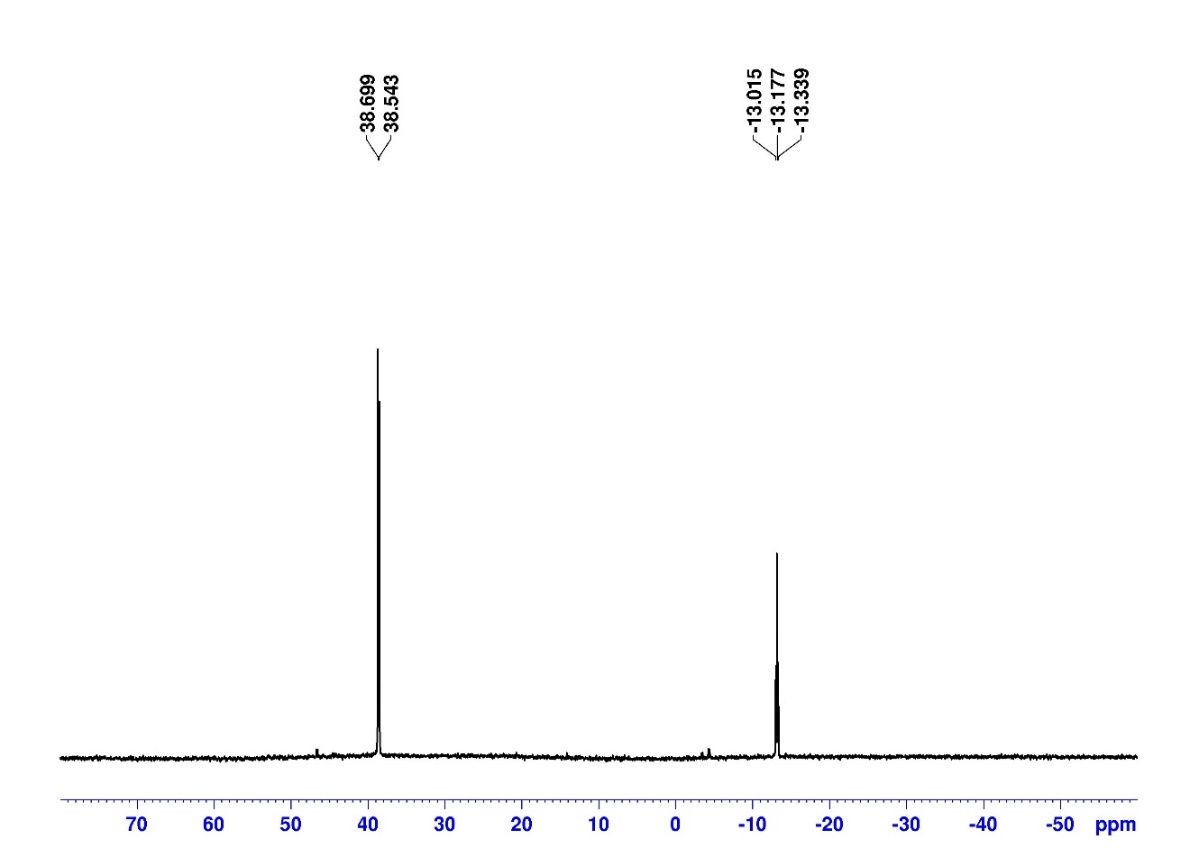
**

**Figure S9**. ^31^P{^1^H} NMR (242.94MHz, DMF-d_7_, 25ºC) of **1**

**
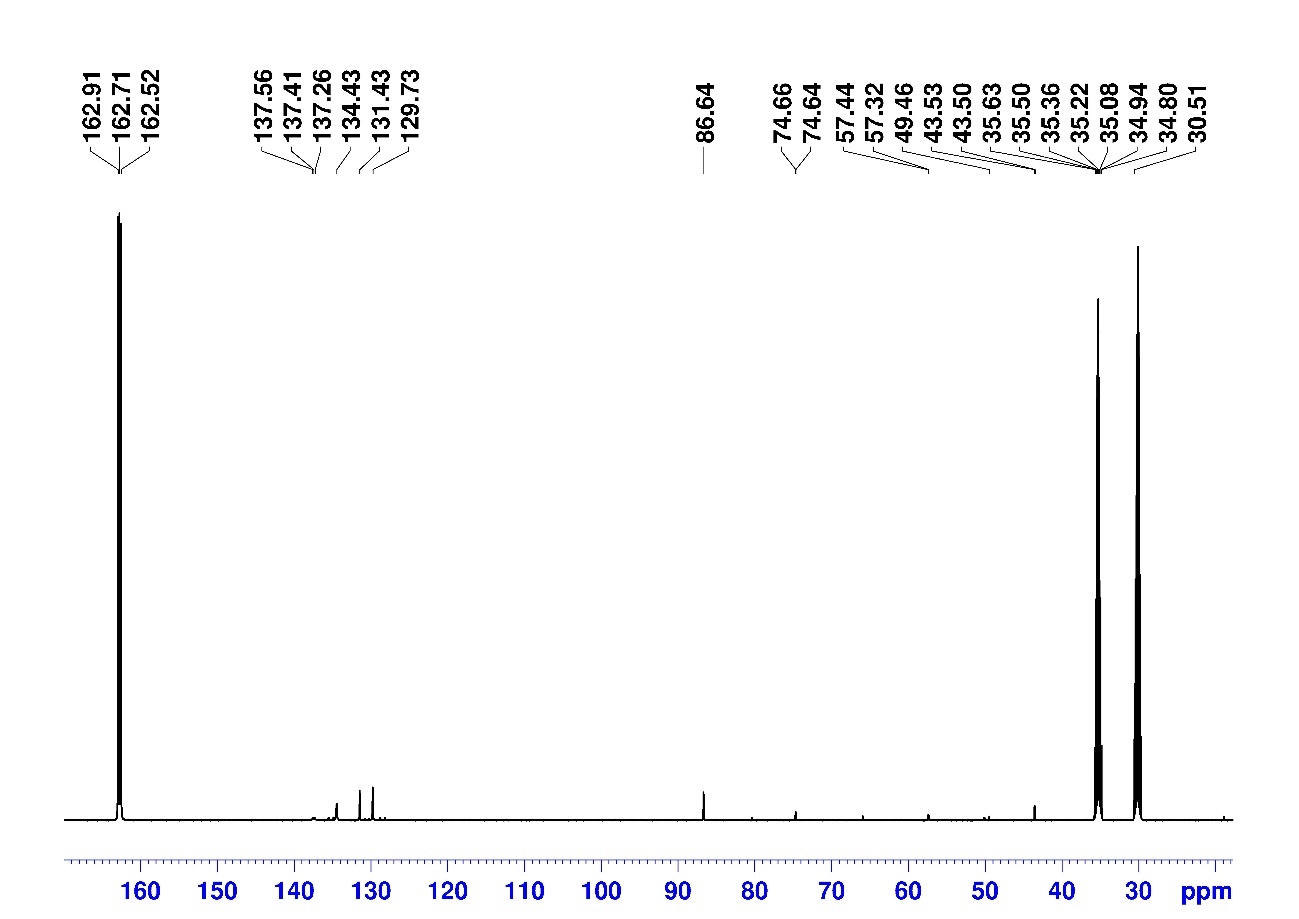
**

**Figure S10**. ^13^C{^1^H} NMR (150.90MHz, DMF-d_7_, 25ºC) of **1**

**
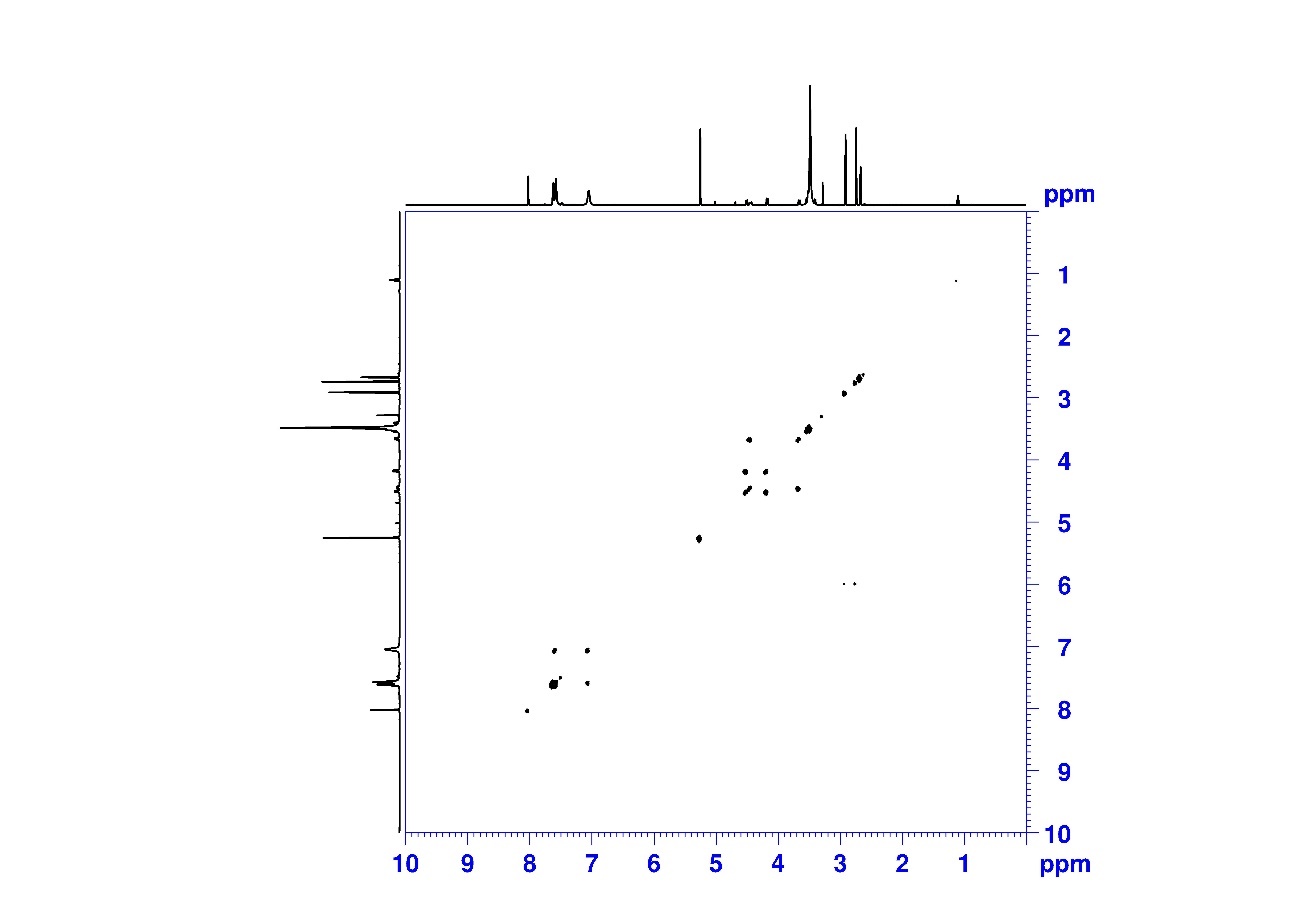
**

**Figure S11**. ^1^H-^1^H COSY NMR (600.13MHz, 600.13MHz, DMF-d_7_, 25ºC) of **1**


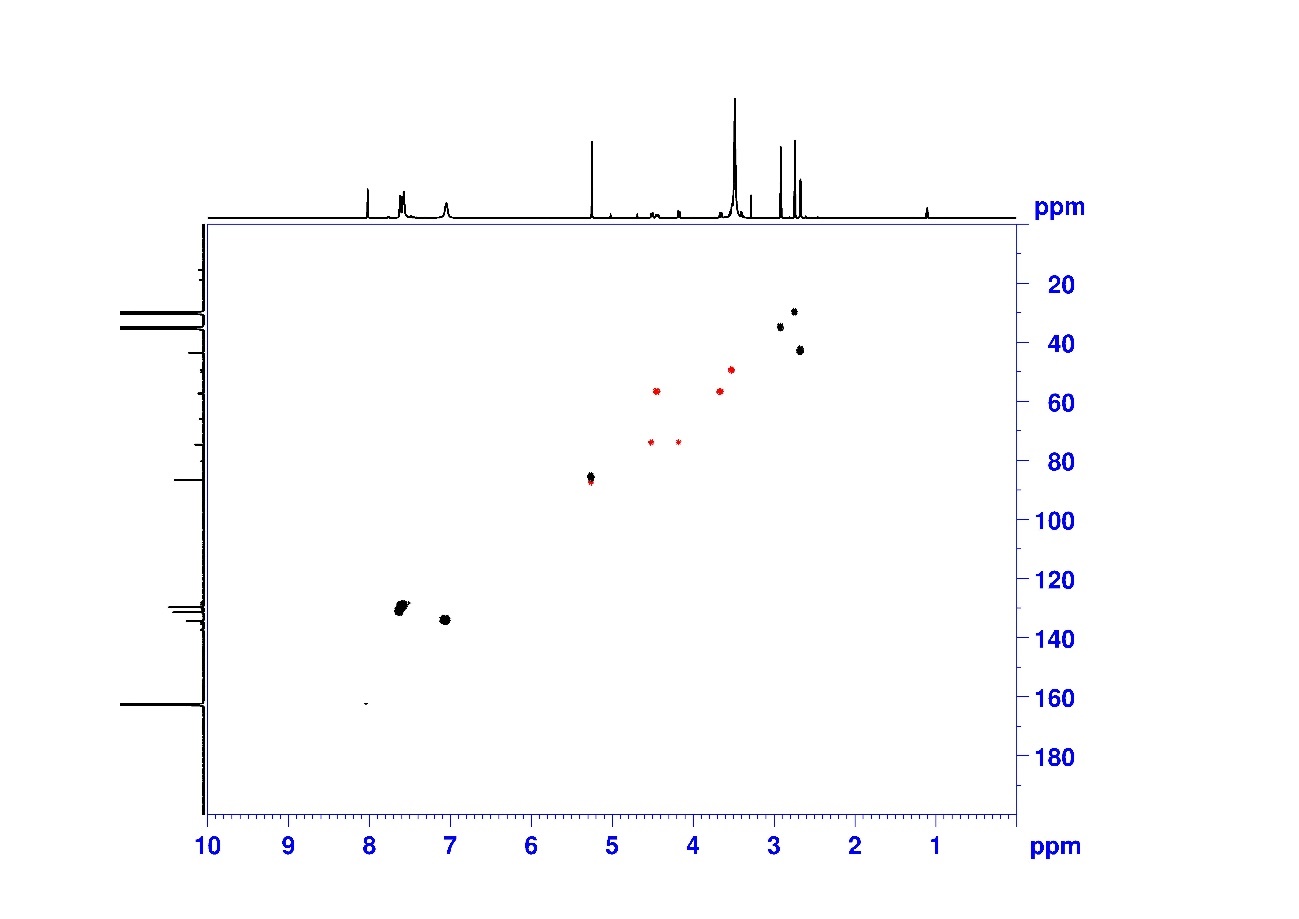


**Figure S12**. ^1^H-^13^C HSQC NMR (600.13MHz, 150.90MHz, DMF-d_7_, 25ºC) of **1**

**
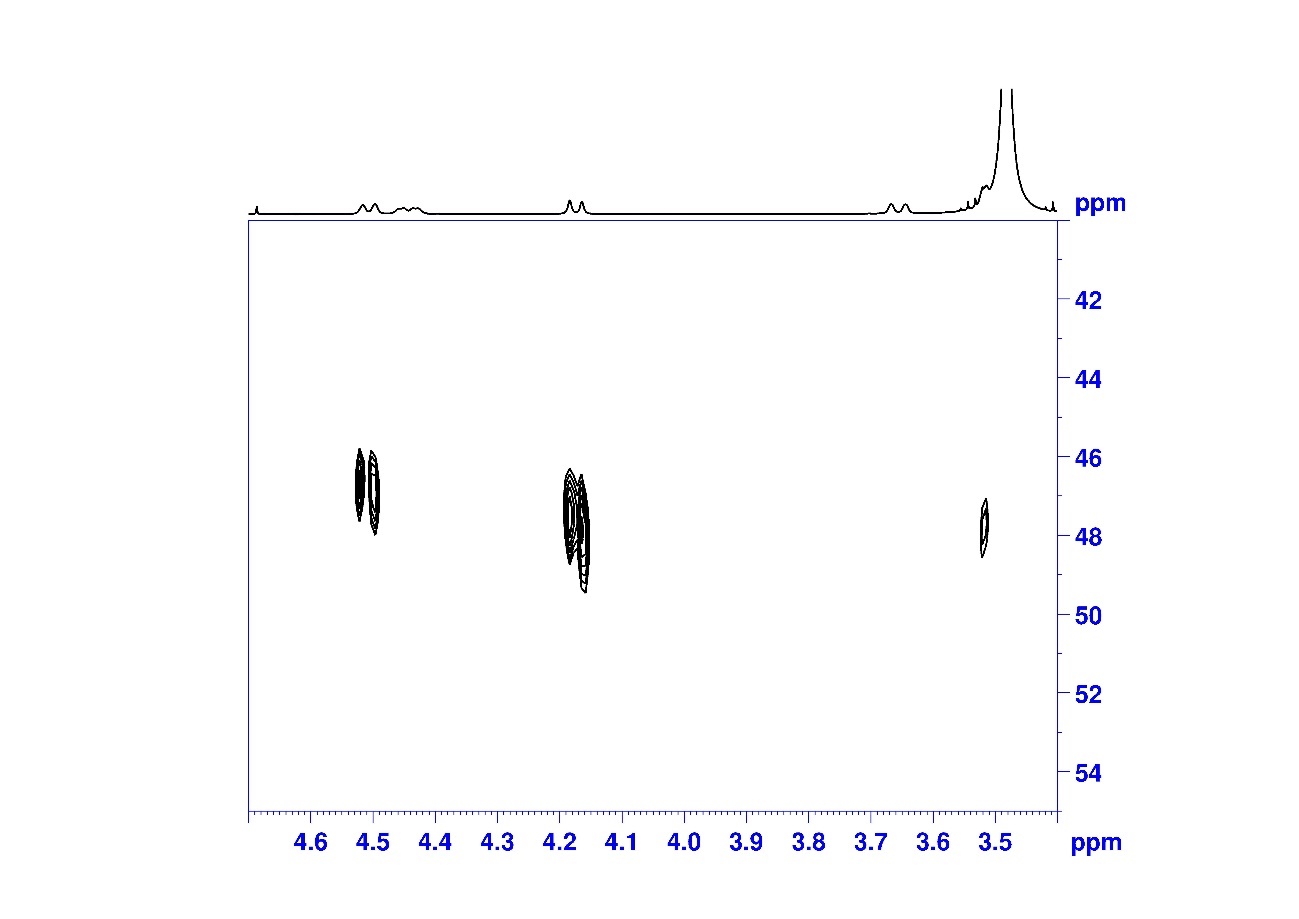
**

**Figure S13**. ^1^H-^15^N HMBC NMR (600.13MHz, 60.81MHz, DMF-d_7_, 25ºC) of **1**

**
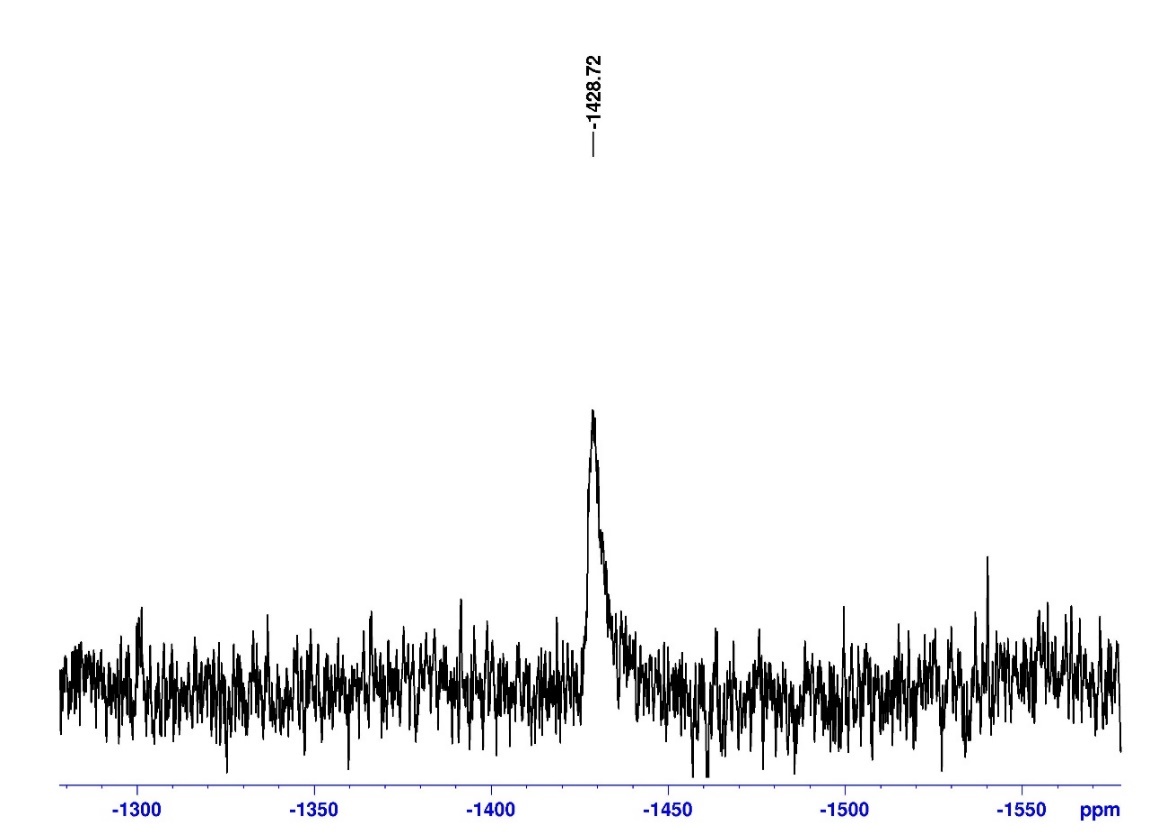
**

**Figure S14**. ^195^Pt{^1^H} NMR (107.51 MHz, DMF-d_7_, 25ºC) of **1**

**
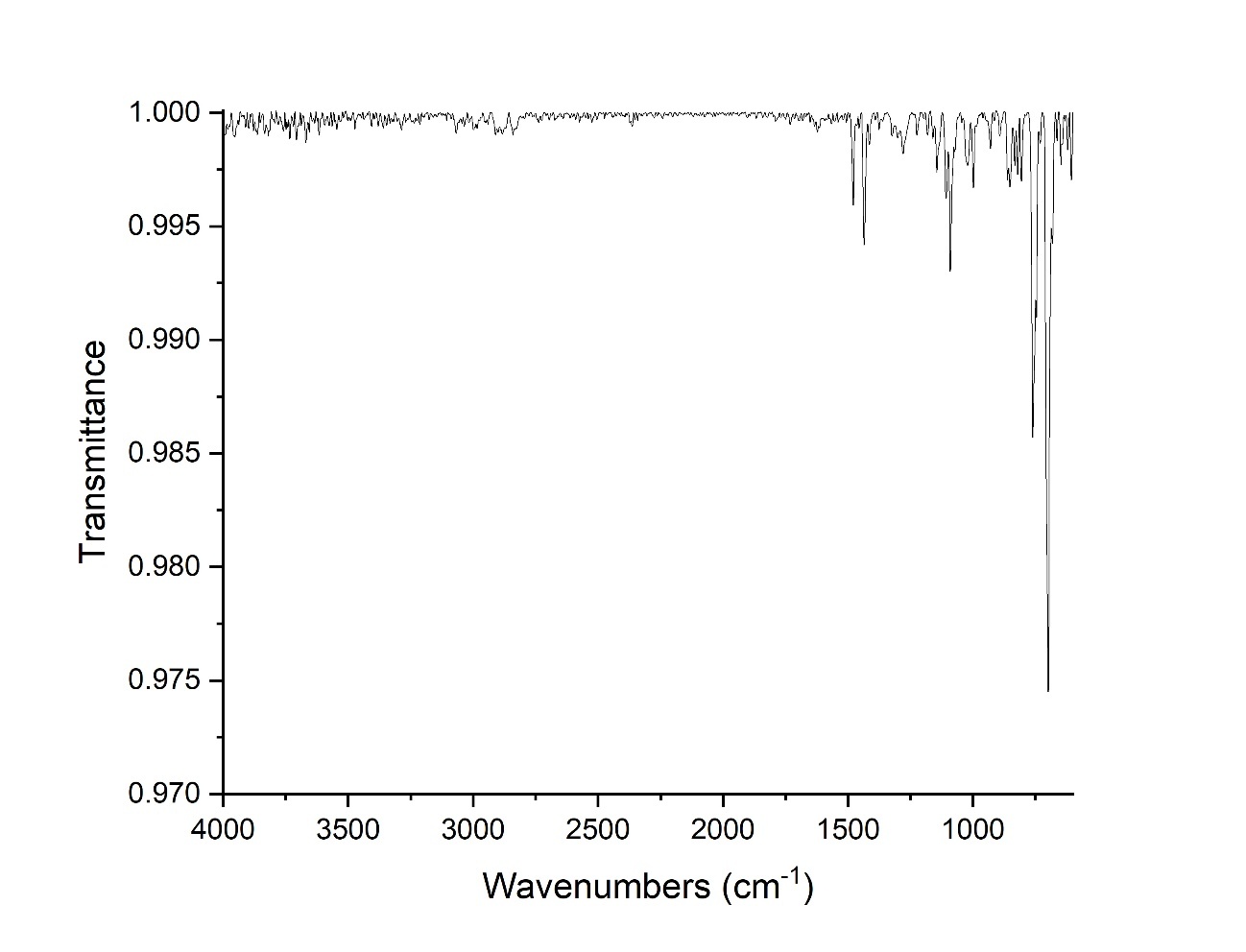

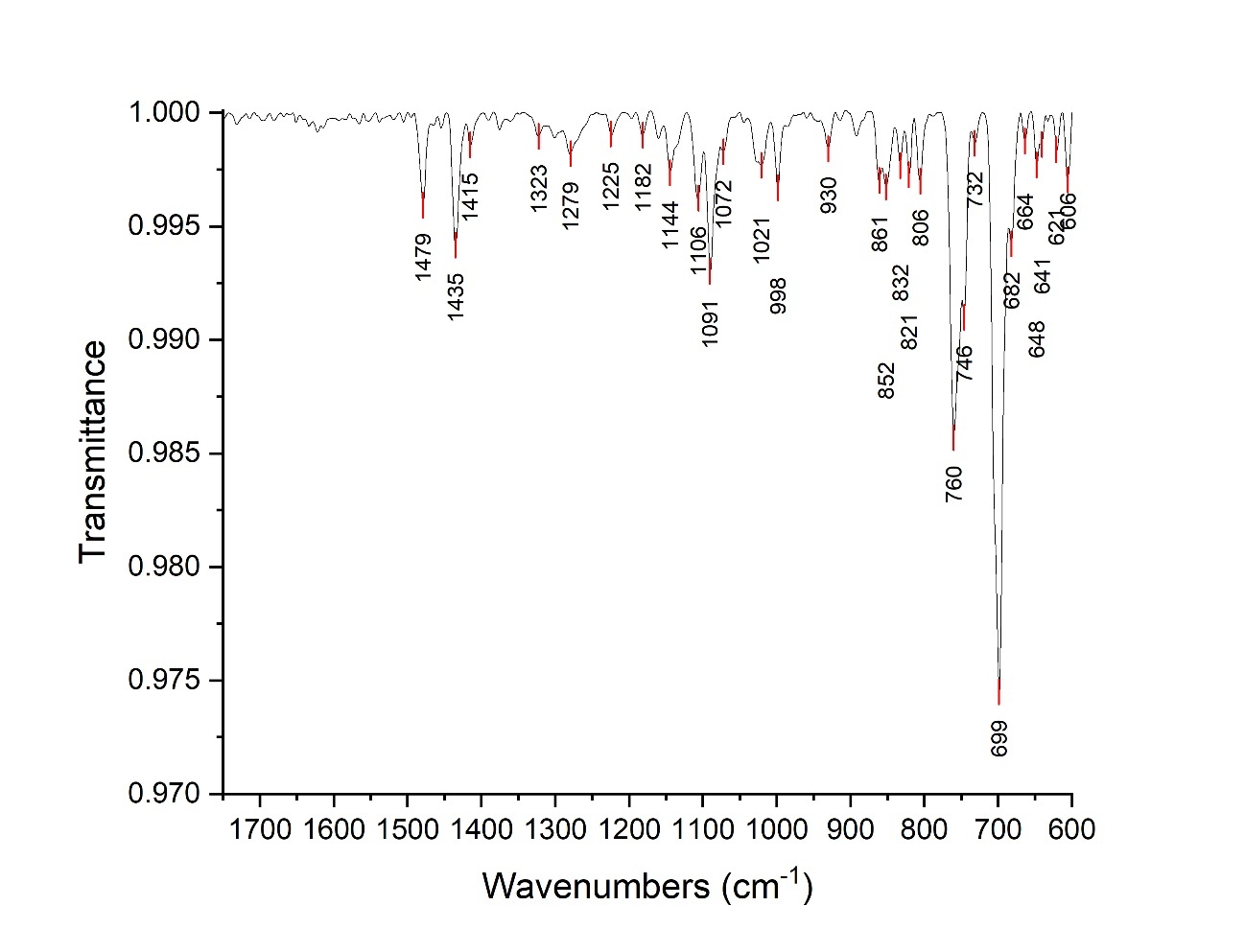
**

**Figure S15**. IR (ATR) of **1** and ampliation in the 1750-600 cm^-1^ region.

### **Synthesis of [RuCp(PPh_3_)_2_-µ-dmoPTA-1ĸ*P*:2ĸ^2^*N,N’*-Pt(DMF- ĸ^2^*C,O*)][PtCl_4_] (2)**

A saturated solution of **1** (77.7 mg, 0.064 mmol) in DMF was stored at 37ºC for 24 h affording dark yellow crystals. Yield: 51.3 mg, 54%. C_51_H_57_Cl_4_N_4_OP_3_Pt_2_Ru (PM = 1467.98 g·mol^-1^). Calcd: C 41.74; H 3.92; N 3.82. Found: C 41.55; H 4.06; N 3.69. IR (ATR, cm^-1^): 618(m), 640(m), 669(w), 698(s), 749(m), 807(w), 831(m), 876(w), 922(w), 980(w), 1030(m), 1059(m), 1093(m), 1133(m), 1159(m), 1184(m), 1225(w), 1256(m), 1357(w), 1383(w), 1416(w), 1434(m), 1455(w), 1665(m), 2840(w), 2883(w), 2895(w), 3051(w).


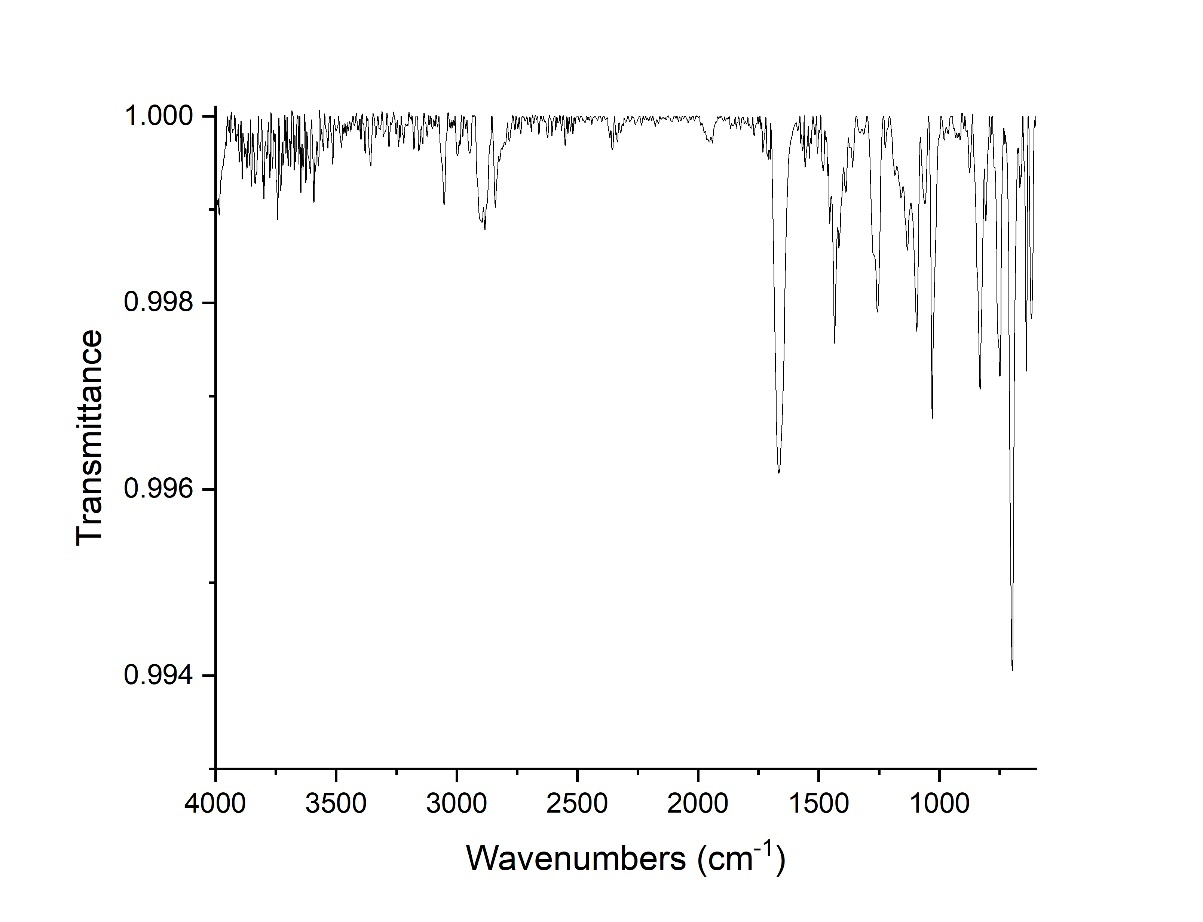


**
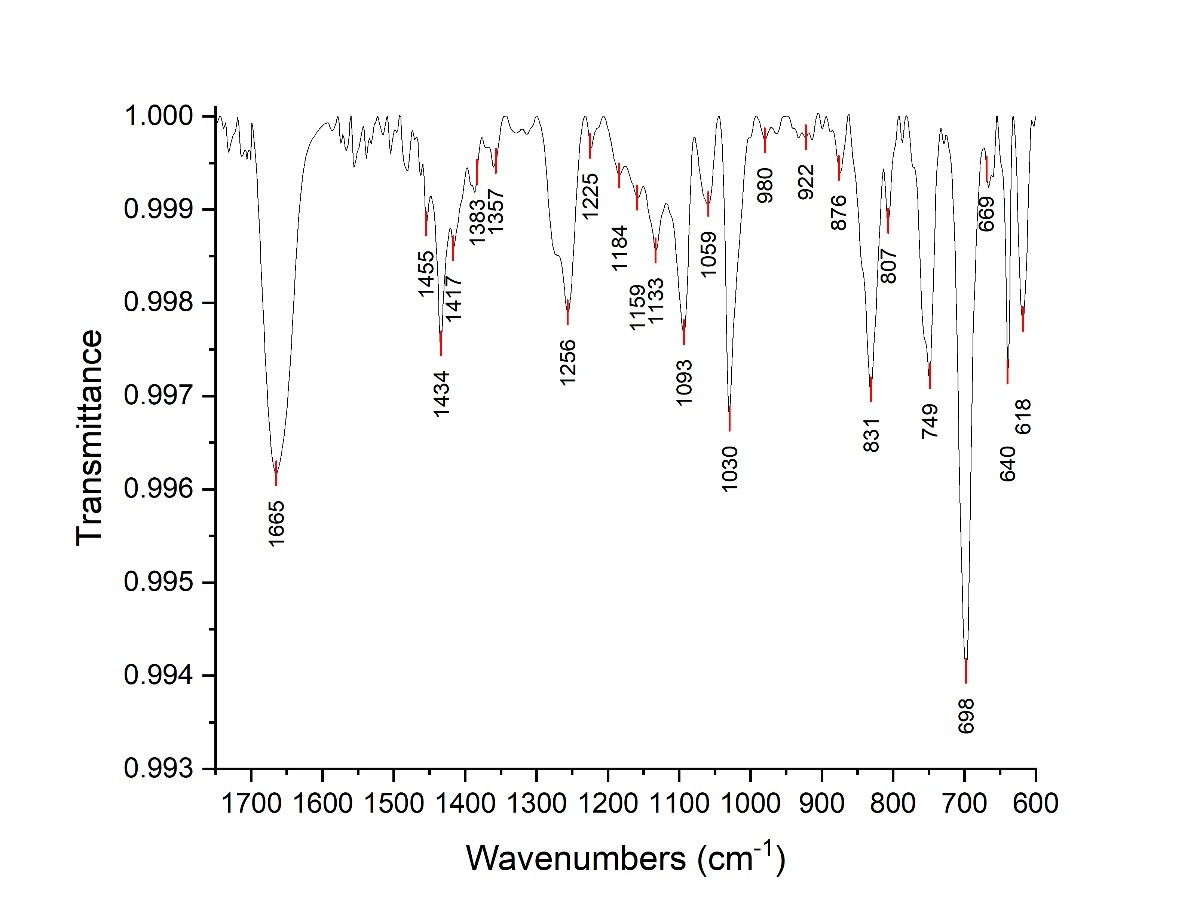
**

**Figure S16**. IR (ATR) of **2** and ampliation in the 1750-600 cm^-1^ region.

### **Synthesis of [RuClCp(HdmoPTA)(PPh_3_)]_2_[PtCl_4_] (3)**

A solution of K_2_[PtCl_4_] (26.4 mg, 0.064 mmol) in H_2_O (200 µL) was added dropwise into a solution of [RuClCp(PPh_3_)(HdmoPTA-ĸ*P*)](CF_3_SO_3_) (50 mg, 0.064 mmol) in dry MeOH (10 mL). After 30 minutes of stirring, a light-orange precipitate is obtained. This precipitate was suction filtered, washed with Et_2_O (3 x 10 mL) and dried under vacuum. Yield: 40.3 mg, 78%, C_30_H_37_Cl_3_N_3_P_2_Pt_0.5_Ru (PM = 806.54 g·mol^-1^). Calc: C 44.69; H 4.63; N 5.22. Found: C 44.41; H 4.32; N 4.97. ^1^H NMR (500.13 MHz, DMSO-d_6_, 25ºC) δ (ppm): 2.25 + 2.38 (6H, s + s, N*CH_3_*), 2.91 + 3.17 + 3.65 + 3.91 (1H + 1H + 1H + 1H, m + m + m + m, P*CH_2_*NCH_3_), 3.08 (2H, m, P*CH_2_*N), 3.80 + 4.24 (2H + 2H, m + m, N*CH_2_*N), 4.52 (5H, s, Cp), 7.45 (15H, m, aromatics, PPh_3_). ^13^C{^1^H} NMR (125.76 MHz, DMSO-d_6_, 25ºC) δ (ppm): 43.28 (m, N*CH_3_*), 47.70 (P*CH_2_*N), 54.84 (m, P*CH_2_*NCH_3_), 74.90 (m, N*CH_2_*N), 79.58 (s, Cp), 128.60-134.72 (m, PPh_3_). ^31^P{^1^H} NMR (202.46 MHz, DMSO-d_6_, 25ºC) δ (ppm): 46.26 (d, ^2^*J*_pp_ = 43.8 Hz, PPh_3_), -4.15 (d, ^2^*J*_PP_ = 43.5 Hz, HdmoPTA). ^195^Pt{^1^H} NMR (107.51MHz, DMSO-d_6_, 25ºC) δ (ppm): -2956.71 ([PtCl_3_(DMSO-κ*S*)]^-^). IR (ATR, cm^-1^): 610(m), 668(w), 684(w), 699(s), 753(m), 801(m), 851(m), 891(w), 912(w), 930(w), 986(w), 997(w), 1031(w), 1047(w), 1089(m), 1100(w), 1115(w), 1137(w), 1152(w), 1184(w), 1222(w), 1257(w), 1272(w), 1293(w), 1318(w), 1351(w), 1361(w), 1371(w), 1415(w), 1430(w), 1456(w), 1479(w), 2746(w), 2951(w), 2842(w), 2917(w), 2799(w).

**
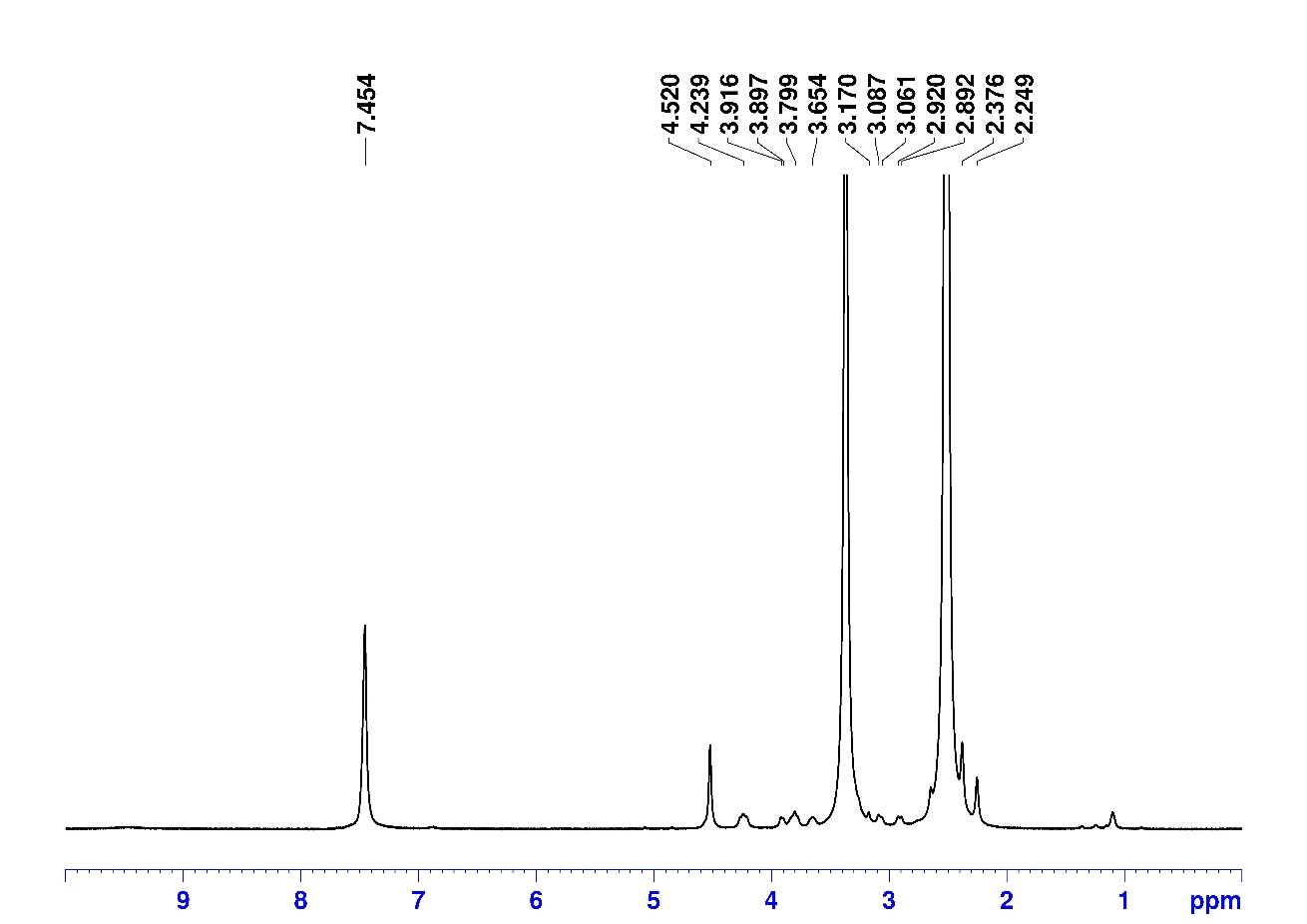
**

**Figure S17**. ^1^H NMR (500.13MHz, DMSO-d_6_, 25ºC) of **3**

**
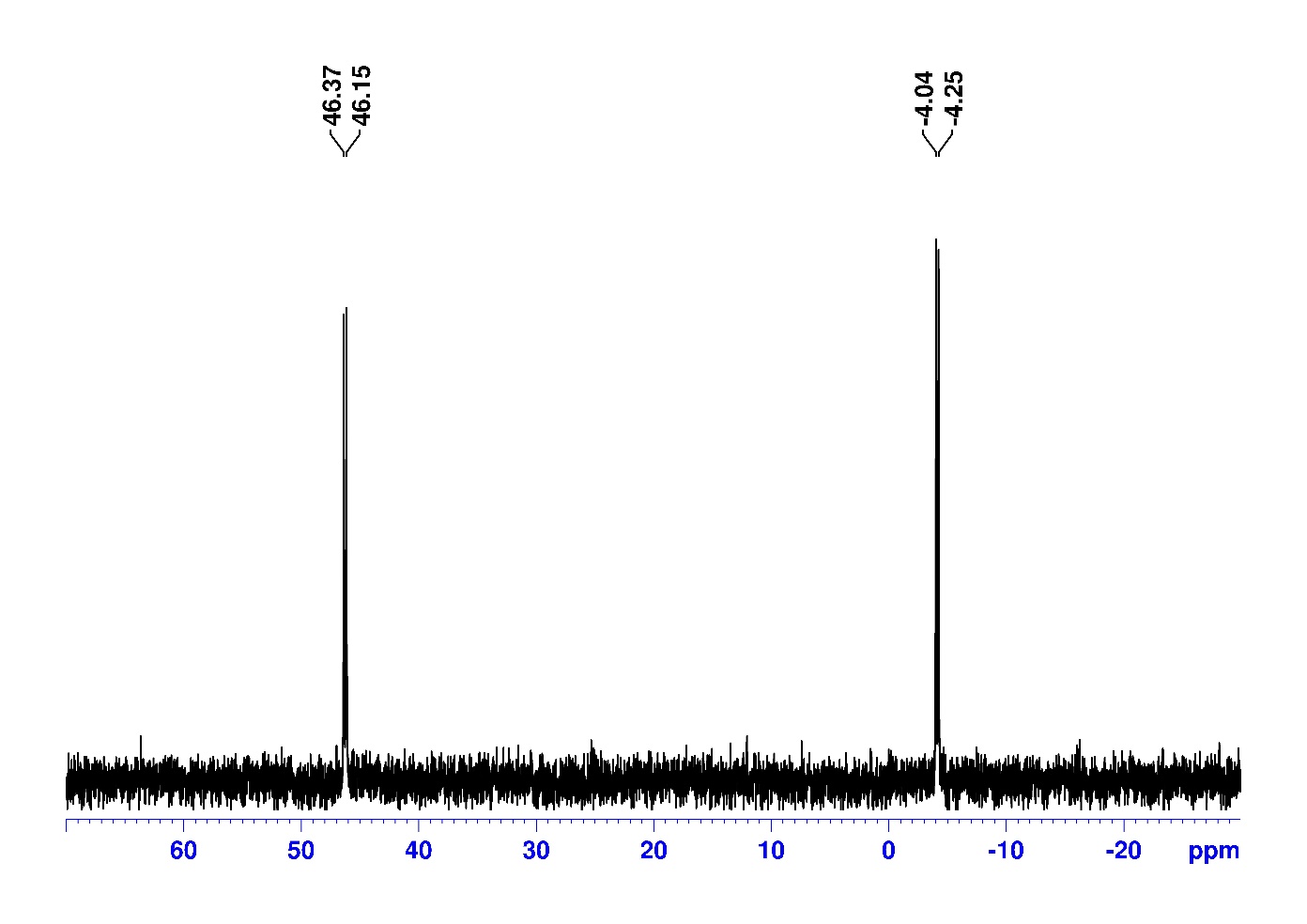
**

**Figure S18**. ^31^P{^1^H} NMR (242.94MHz, DMSO-d_6_, 25ºC) of **3**

**
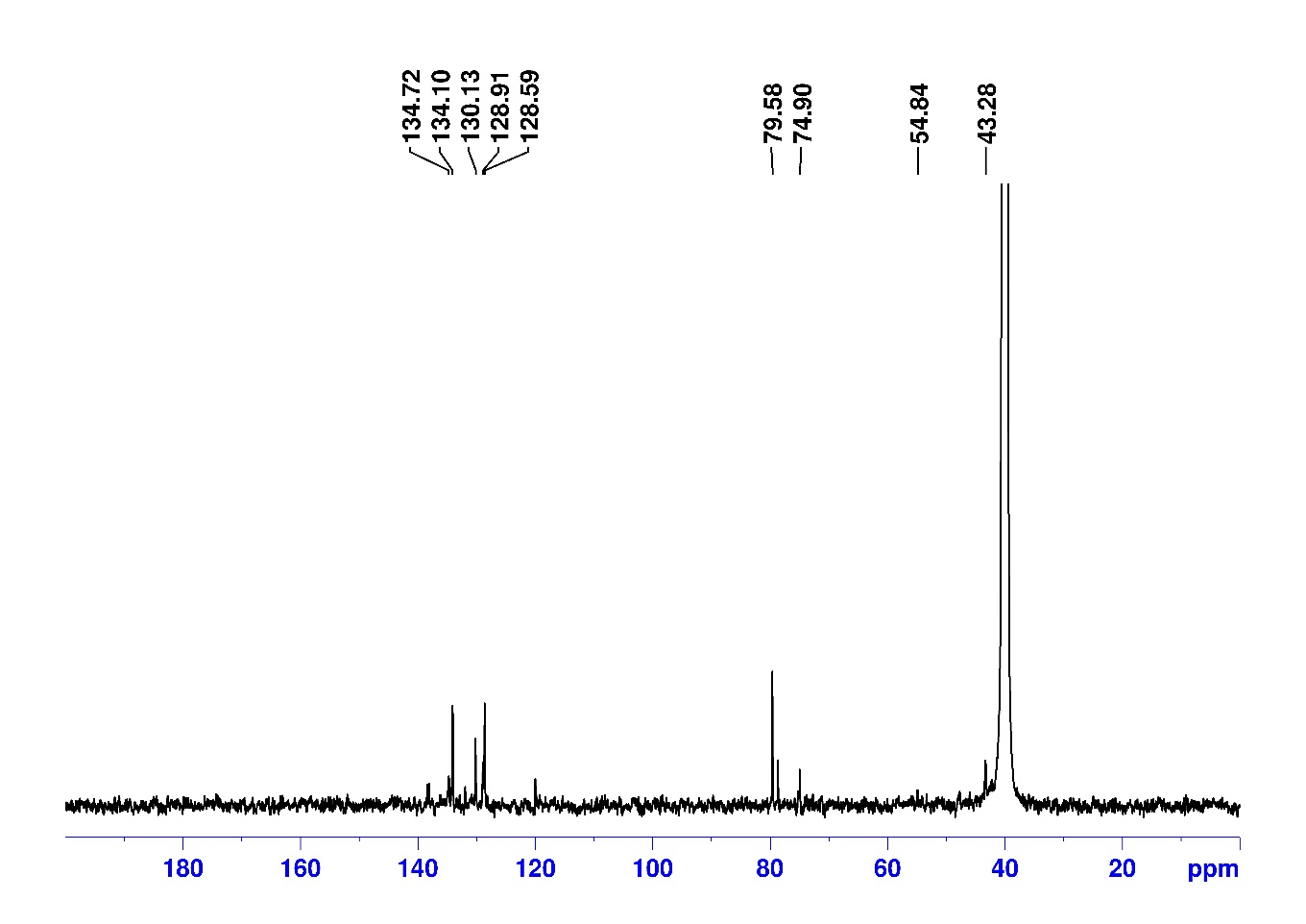
**

**Figure S19**. ^13^C{^1^H} NMR (125.76MHz, DMSO-d_6_, 25ºC) of **3**

**
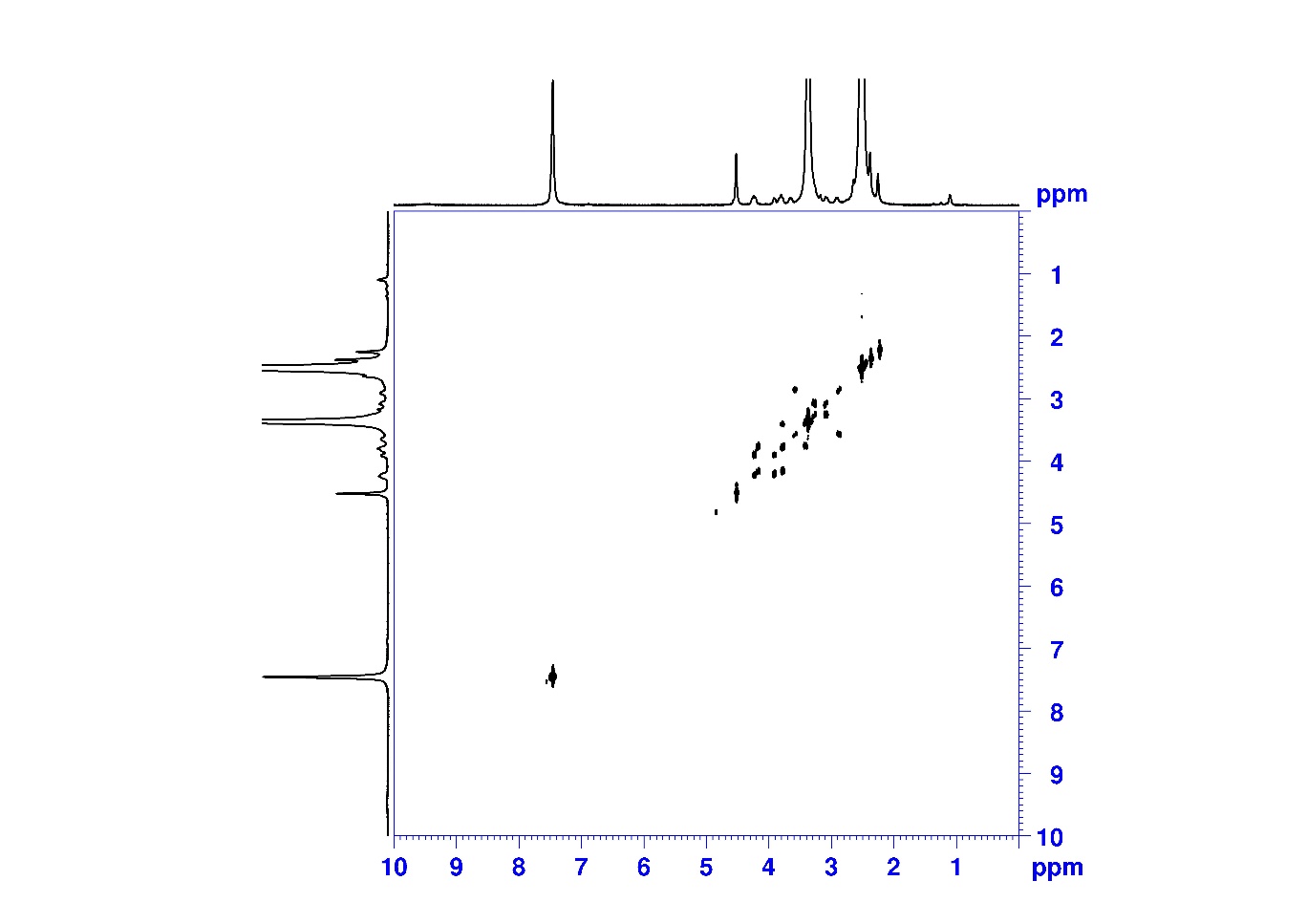
**

**Figure S20**. ^1^H-^1^H COSY NMR (500.13MHz, 500.13MHz, DMSO-d_6_, 25ºC) of **3**


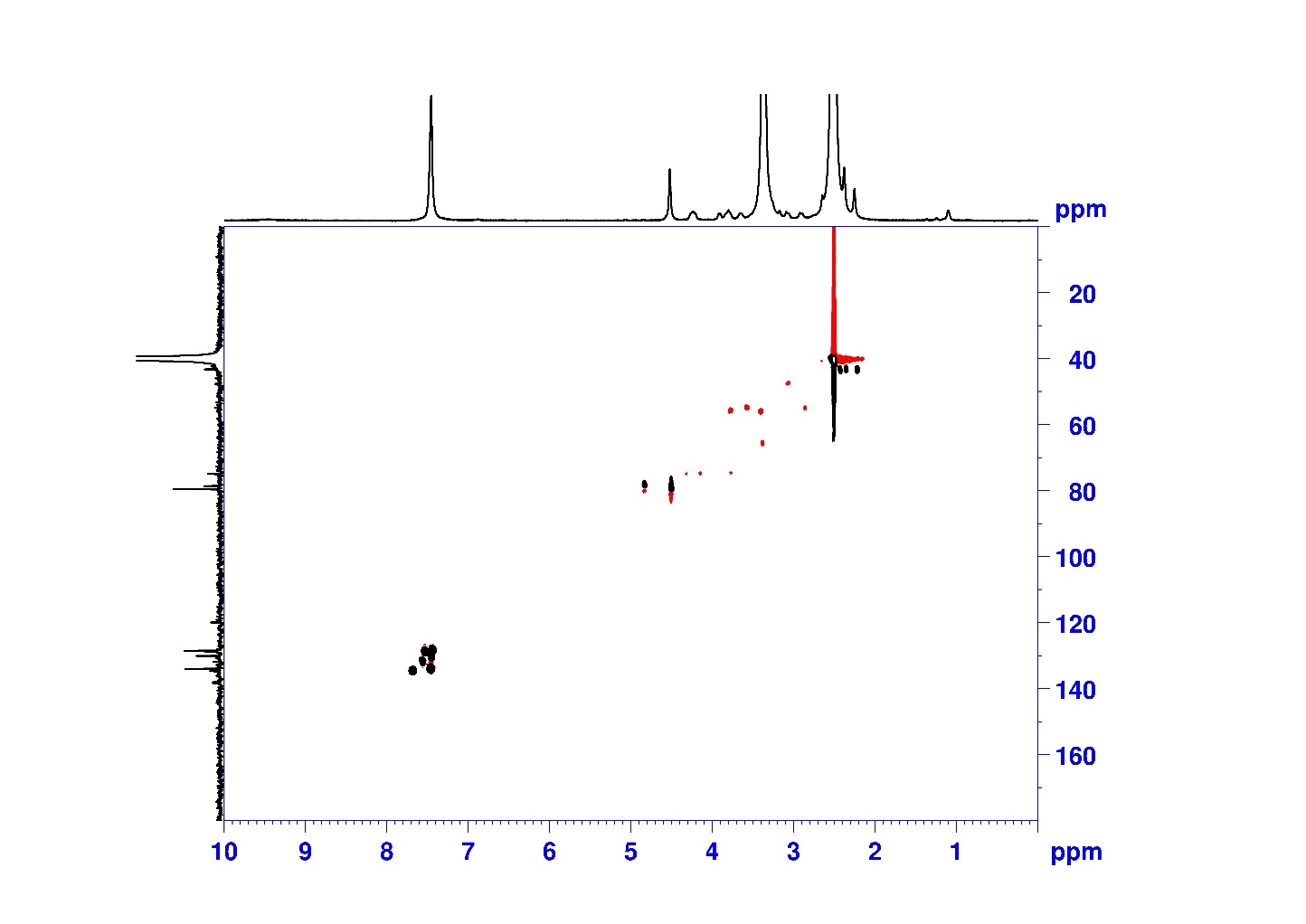


**Figure S21**. ^1^H-^13^C HSQC NMR (500.13MHz, 125.76MHz, DMSO-d_6_, 25ºC) of **3**.

**
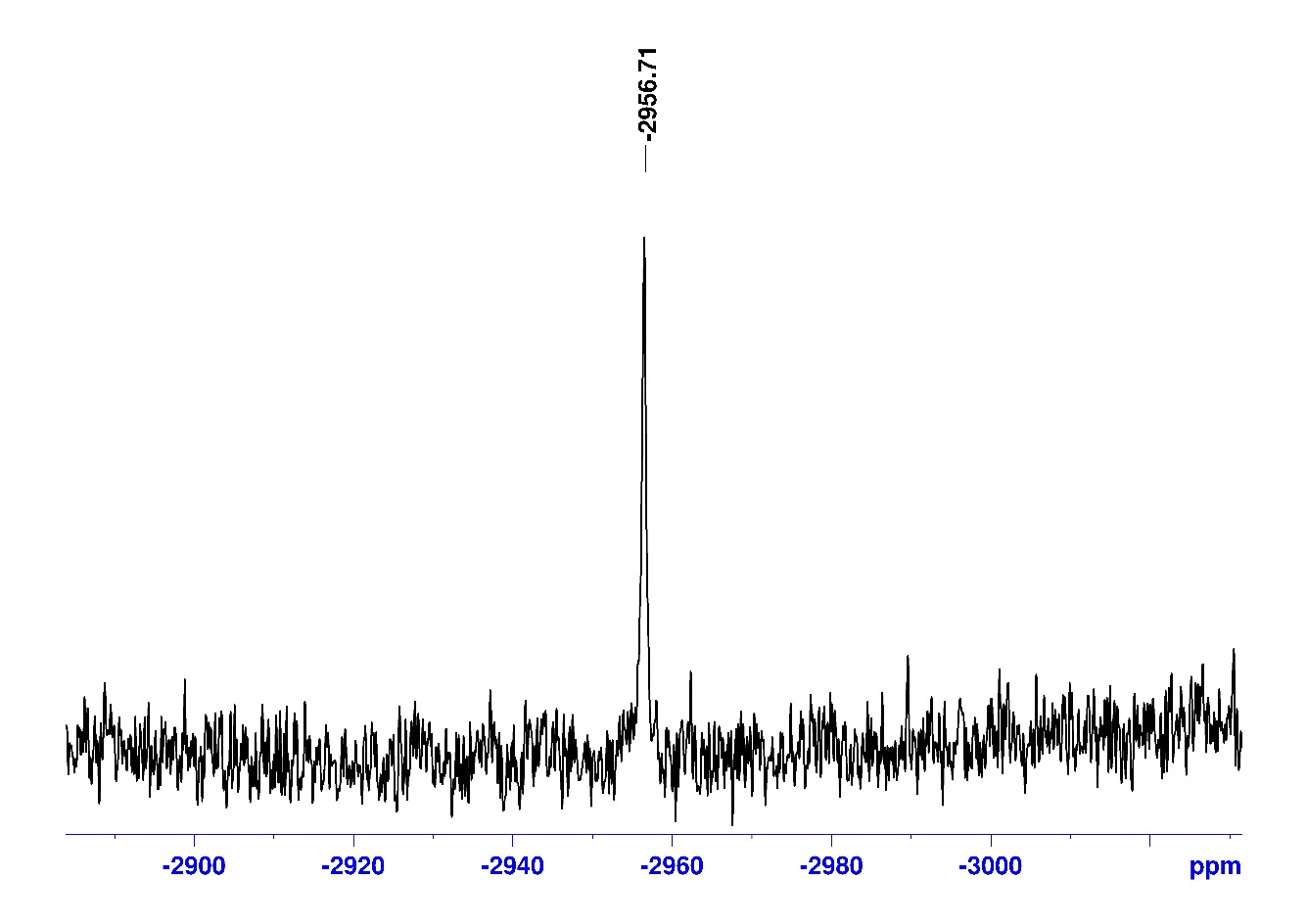
**

**Figure S22**. ^195^Pt{^1^H} NMR (107.51 MHz, DMSO-d_6_, 25ºC) of **3.**

**
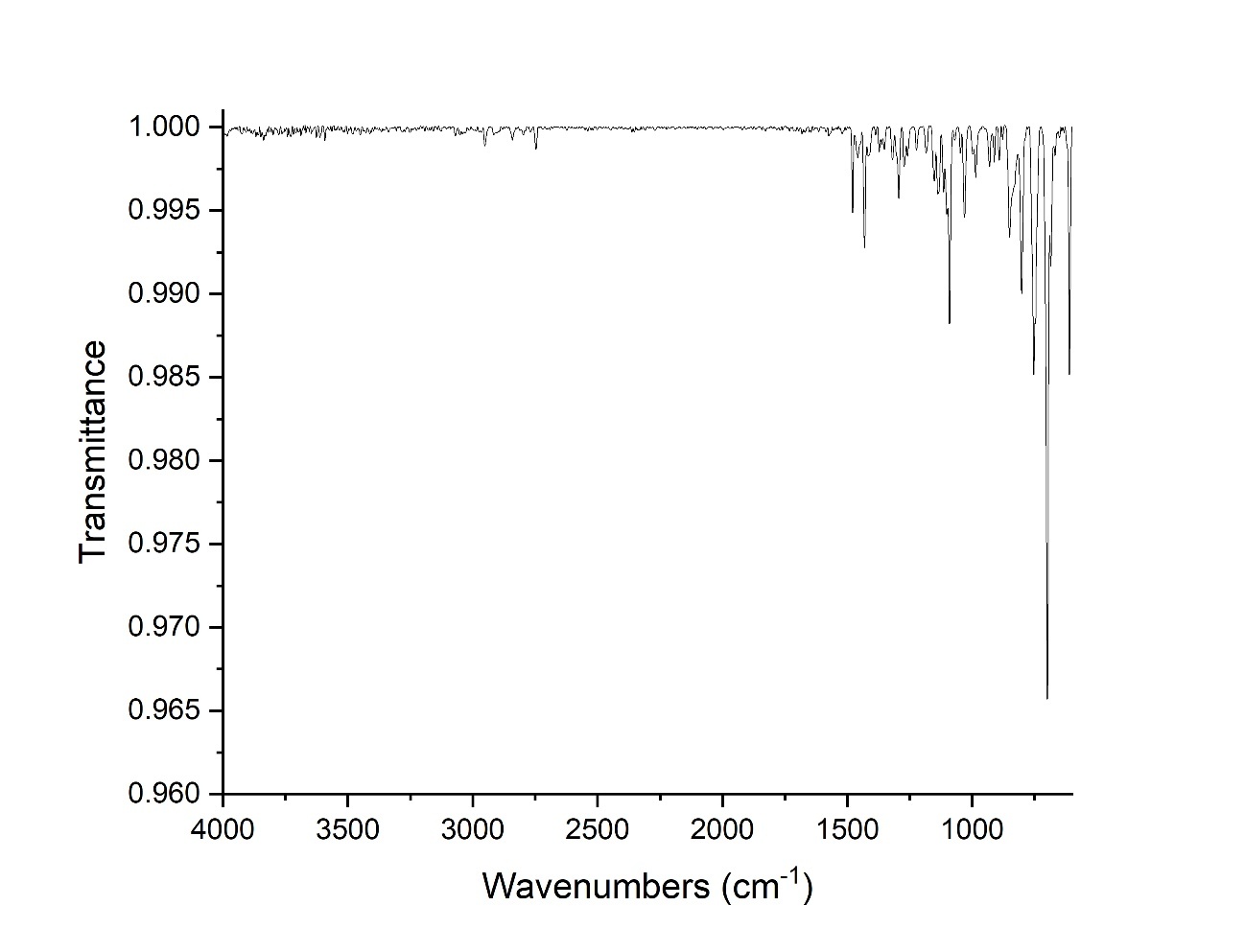

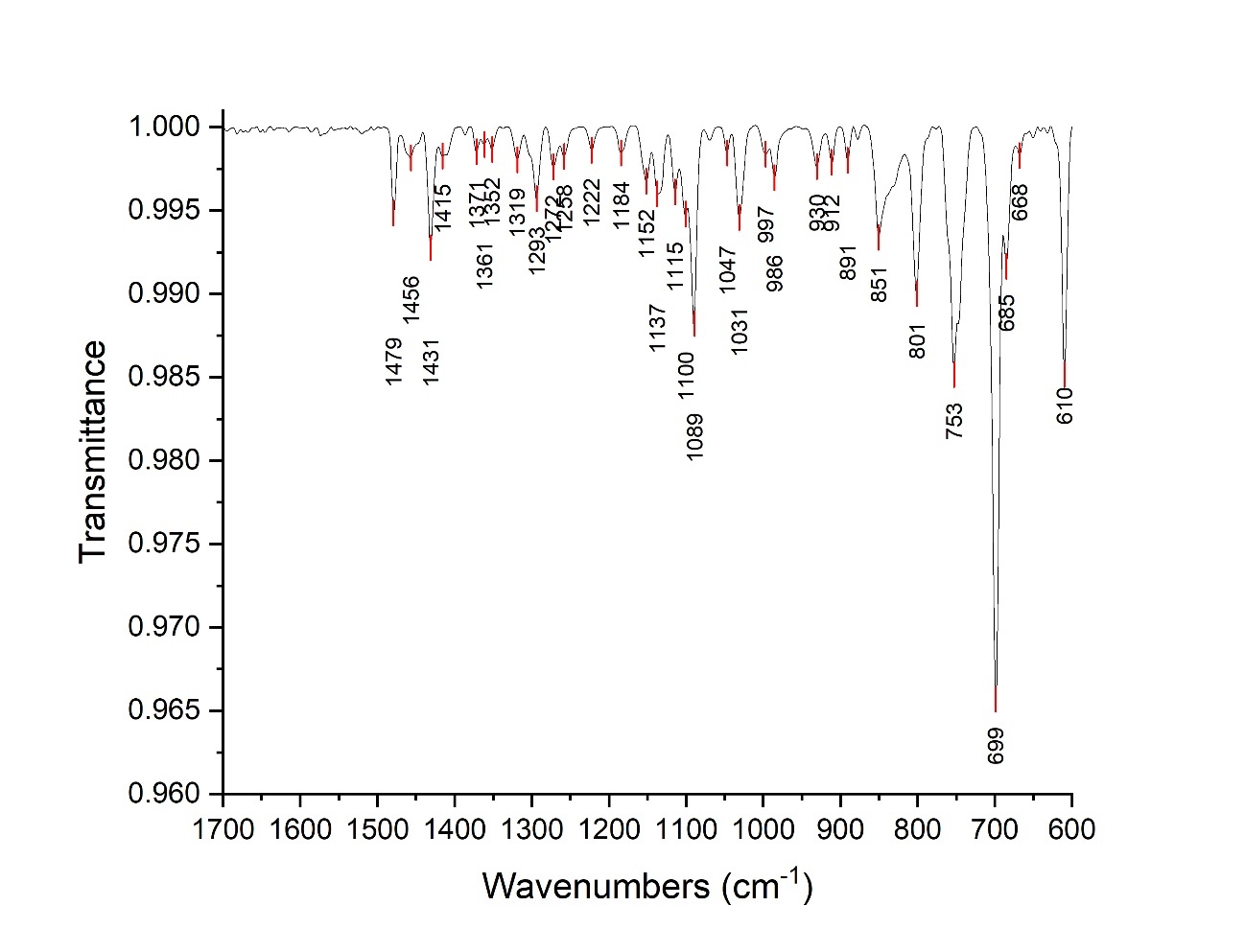
**

**Figure S23**. IR (ATR) of **3** and ampliation in the 1700-600 cm^-1^ region.

### **Synthesis of [RuClCp(PPh_3_)-µ-dmoPTA-1ĸ*P*:2ĸ^2^*N,N’*-Pt(DMF- ĸ^2^*C,O*)]_2_[PtCl_4_] (4)**

A saturated solution of **3** (56.9 mg, 0.07 mmol) in DMF was stored at 37ºC for 24 h affording a brown precipitate. Yield: 34.1 mg, 45%. C_33_H_42_Cl_3_N_4_OP_2_Pt_1.5_Ru (PM = 1072.7 g·mol^-1^). Calc: C 36.96; H 3.95; N 5.23. Found: C 36.75; H 3.77; N 4.98. IR (ATR, cm^-1^): 615(m), 638(m), 667(w), 698(s), 750(m), 788(w), 806(w), 834(s), 874(m), 899(w), 914(w), 921(w), 977(m), 1030(s), 1064(s), 1092(m), 1133(m), 1211(w), 1256(m), 1312(w), 1360(m), 1386(m), 1417(m), 1435(m), 1455(m), 1462(m), 1505(w), 1556(w), 1666(m), 2840(w), 2890(w), 2907(w), 2971(w).


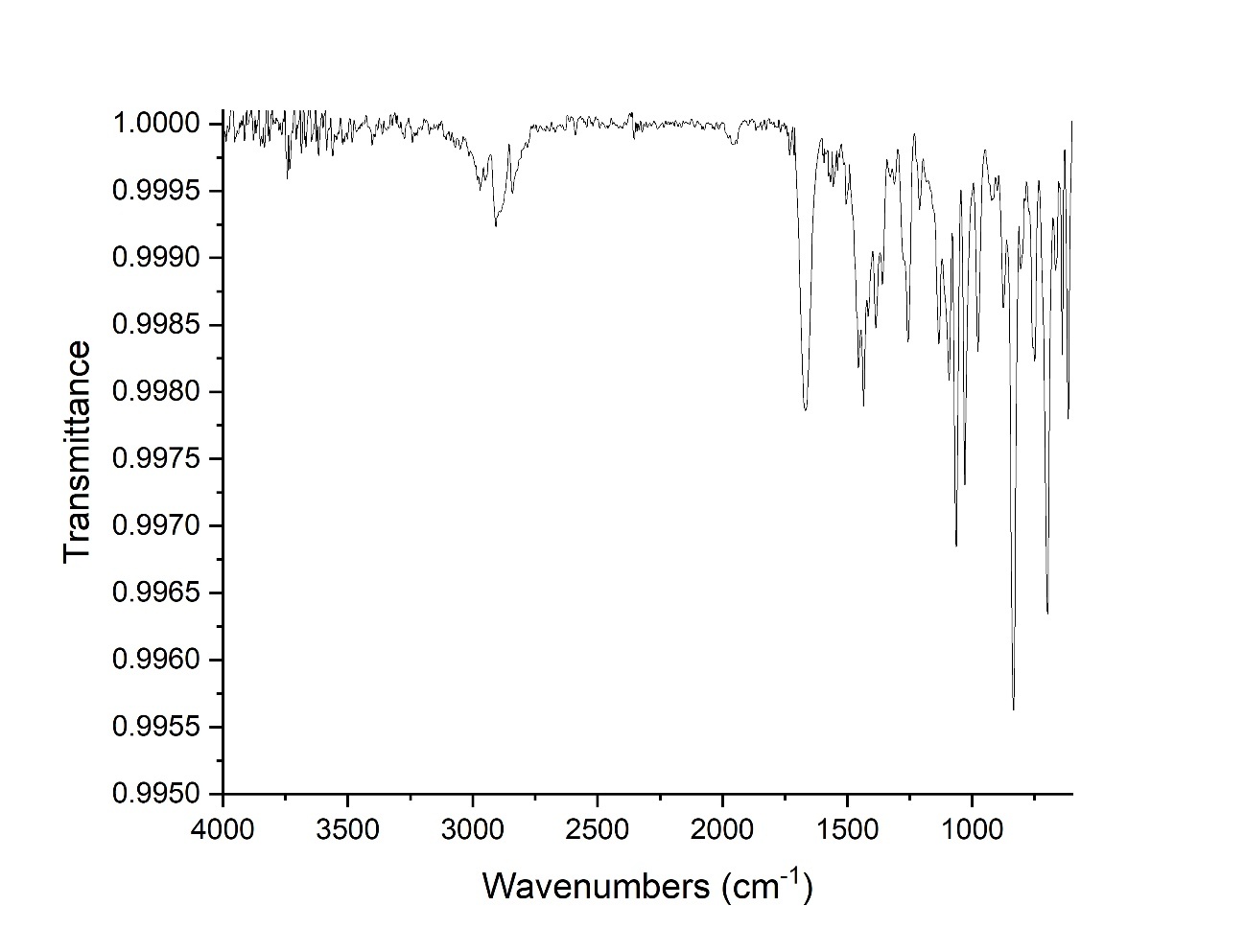


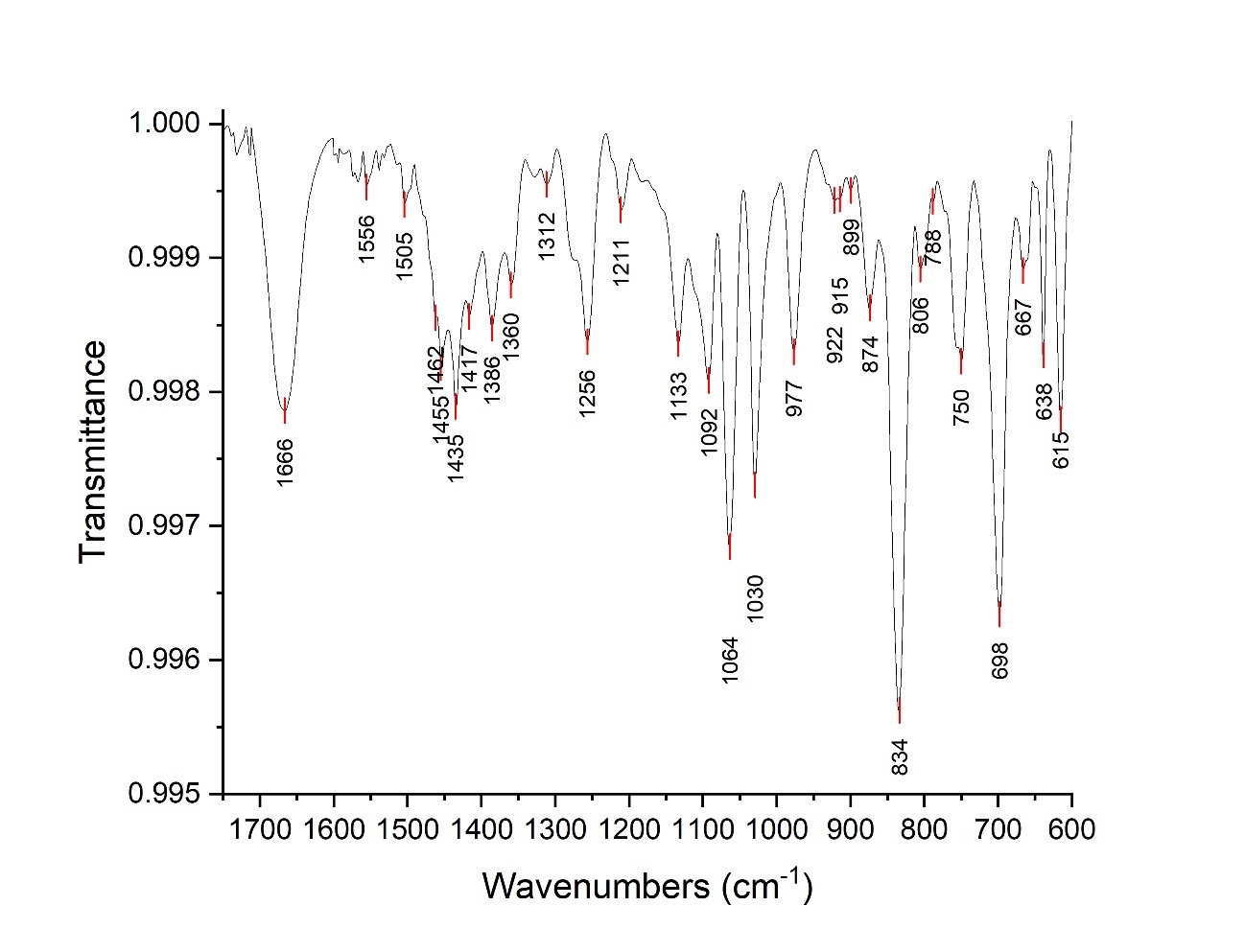


**Figure S24**. IR (ATR) of **4** and ampliation in the 1750-600 cm^-1^ region.

**Stability tests**

The stability of both complexes in DMSO and DMSO/water were performed by introducing the complex (0.01 g) into a 5 mm NMR tube and dissolving it in 0.5 mL of the solvent (DMSO-d_6_ or a mixture of DMSO-d_6_/RPMI 1640 1:1). These solutions were kept at 37ºC and monitored by ^31^P{^1^H} NMR over time.

**Figure S25**. ^31^P{^1^H} NMR (121.49 MHz, DMSO-d_6_, 25ºC) of **1** vs. time


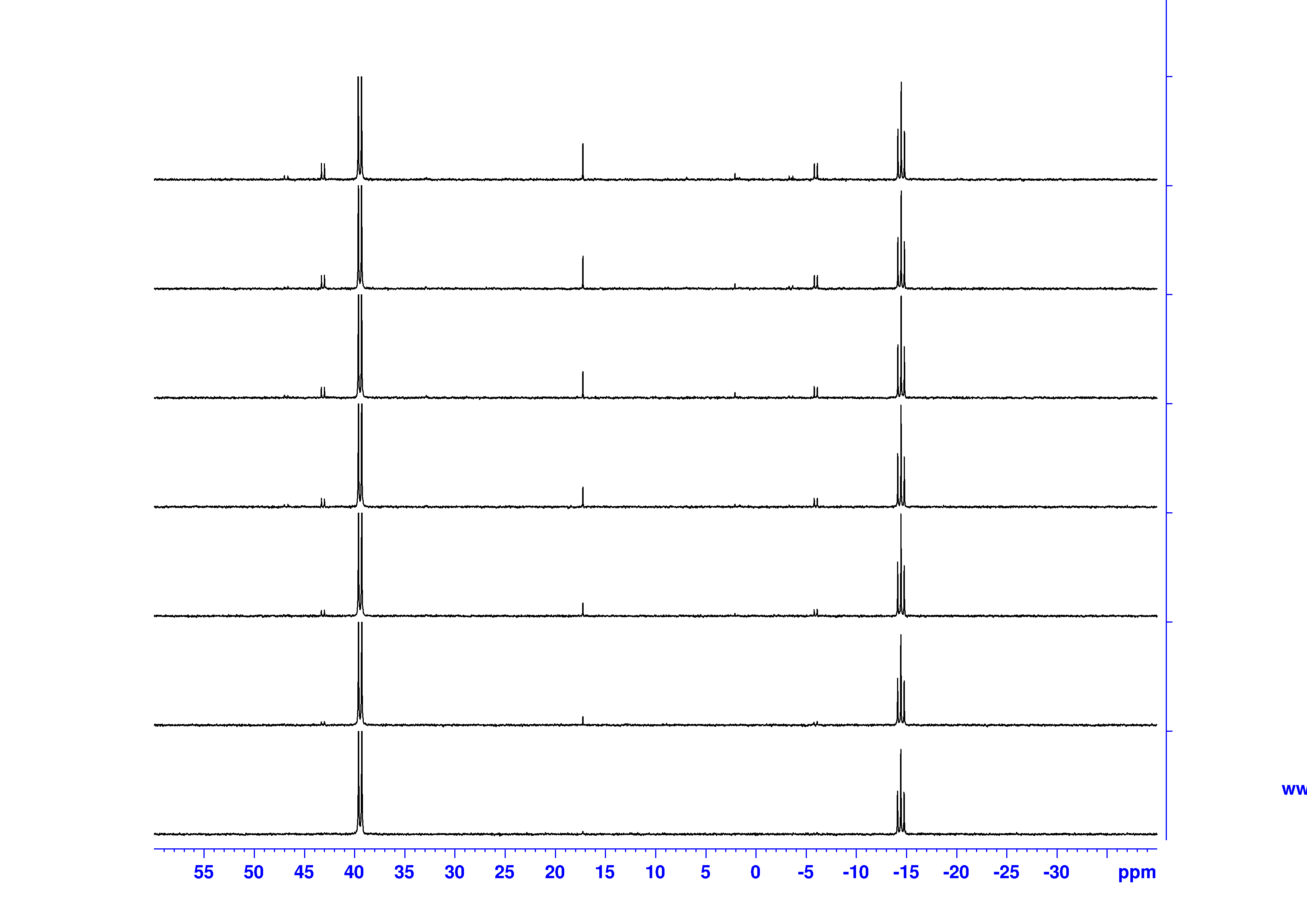


15 min

30 min

1 h

2 h

4 h

24 h

48 h

**Figure S26**. ^31^P{^1^H} NMR (121.49 MHz, DMSO-d_6_/RPMI 1640 1:1, 25ºC) of **1** vs. time.


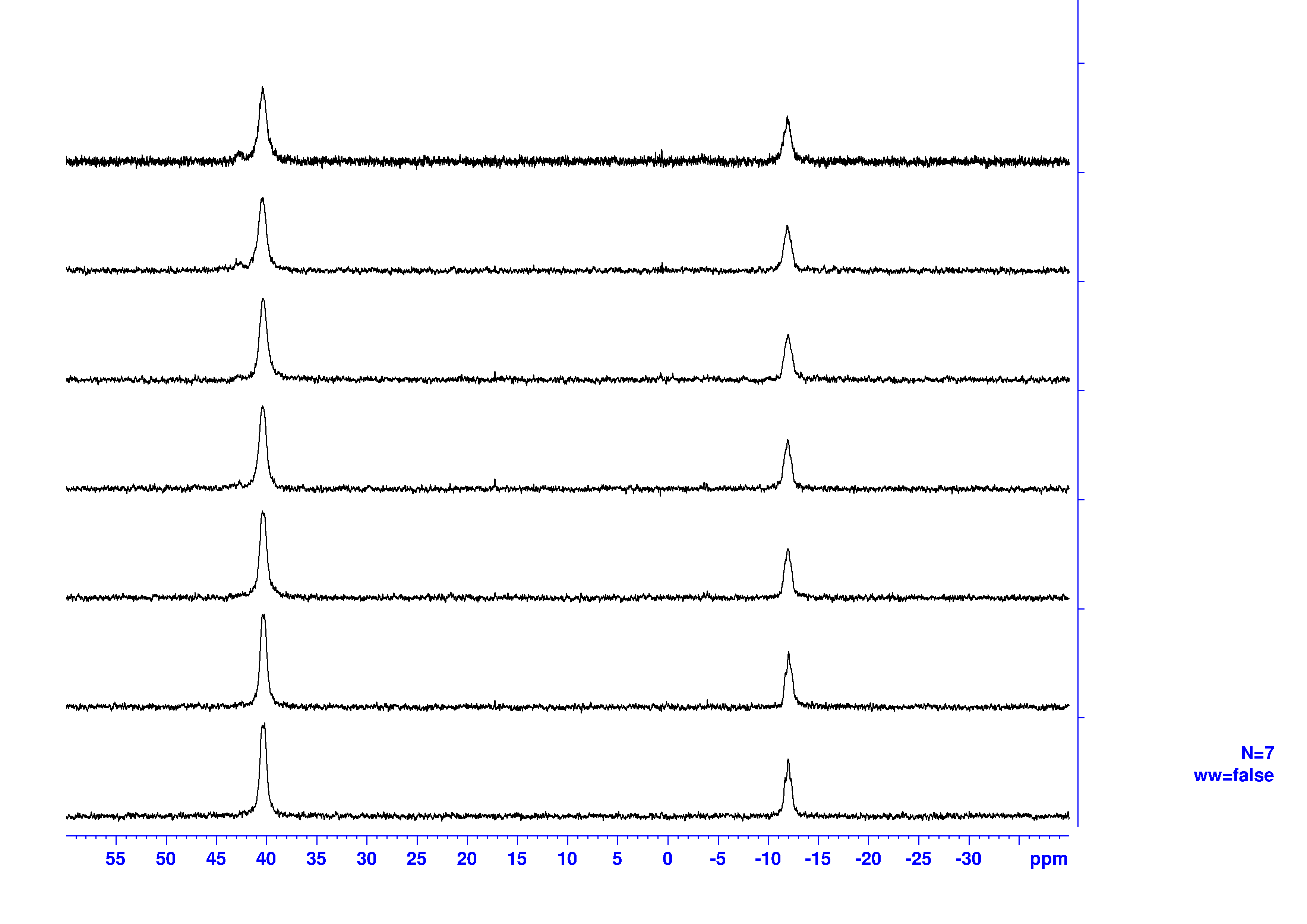


15 min

30 min

1 h

2 h

4 h

24 h

48 h

**Compound cytotoxicity evaluated using the MTT assay**

The antiproliferative tests were performed as previously described.^5,6^

**Table S1**. GI_50_ values (μM) of cisplatin and **1** against human solid tumor cell lines.

|  | Cell lines | | | | | |
| --- | --- | --- | --- | --- | --- | --- |
|  | A549  (Lung) | HBL-100  (Breast) | HeLa  (Cervix) | SW1573  (Lung) | T-47D  (Breast) | WiDr  (Colon) |
| Cisplatin | 4.9±0.2 | 1.9±0.2 | 1.8±0.5 | 2.7±0.4 | 17±3.3 | 23±4.3 |
| **1** | 1.3±0.12 | 0.93±0.12 | 0.08±0.014 | 0.34±0.040 | 2.2±0.29 | 1.4±0.20 |

**Single crystal X-ray diffraction data**

Single crystal X-ray diffraction was performed with a Bruker APEX-II CCD diffractometer at 100K using MoK_α_ (**2**) radiation. Data was integrated (SAINT, Bruker) and scaled (SADABS, Bruker) and finally, the structures were solved with SHELXT^7^ and refined with SHELXL^8^ by least squares. Solution and refinement procedures were accomplished by Olex2 software.^9^ Crystallographic and structural data are given in tables S2-S4. The crystal structure of **2** has been deposited at CSD with CCDC number **2401376**.

| **Table S2**. Crystal data and structure refinement for **2**. | |
| --- | --- |
|  | **2** |
| Empirical formula | C_51_H_57_Cl_4_N_4_OP_3_Pt_2_Ru |
| Formula weight | 1467.96 |
| Temperature/K | 302.10 |
| Crystal system | monoclinic |
| Space group | P2_1_/c |
| a/Å | 15.9131(5) |
| b/Å | 16.5800(5) |
| c/Å | 19.9347(6) |
| α/° | 90 |
| β/° | 103.9160(10) |
| γ/° | 90 |
| Volume/Å^3^ | 5105.2(3) |
| Z | 4 |
| ρ_calc_g/cm^3^ | 1.910 |
| μ/mm^‑1^ | 6.105 |
| F(000) | 2848.0 |
| Crystal size/mm^3^ | 0.117 × 0.048 × 0.022 |
| Radiation | MoKα (λ = 0.71073) |
| 2Θ range for data collection/° | 3.84 to 56.686 |
| Index ranges | -21 ≤ h ≤ 21, -22 ≤ k ≤ 22, -26 ≤ l ≤ 26 |
| Reflections collected | 243663 |
| Independent reflections | 12713 [R_int_ = 0.0664, R_sigma_ = 0.0230] |
| Data/restraints/parameters | 12713/546/592 |
| Goodness-of-fit on F^2^ | 1.085 |
| Final R indexes [I>=2σ (I)] | R_1_ = 0.0580, wR_2_ = 0.1438 |
| Final R indexes [all data] | R_1_ = 0.0743, wR_2_ = 0.1535 |
| Largest diff. peak/hole / e Å^-3^ | 1.90/-2.27 |

| **Table S3**. Bond Lengths for **2**. | | | | | |
| --- | --- | --- | --- | --- | --- |
| **Atom** | **Atom** | **Length/Å** | **Atom** | **Atom** | **Length/Å** |
| Pt1 | N2 | 2.150(8) | C11 | C12 | 1.383(12) |
| Pt1 | O1 | 2.032(7) | C11 | C16 | 1.420(13) |
| Pt1 | N1 | 2.040(9) | C17 | C18 | 1.402(12) |
| Pt1 | C10 | 2.066(11) | C17 | C22 | 1.394(12) |
| Pt2 | Cl4 | 2.287(3) | C35 | C36 | 1.381(12) |
| Pt2 | Cl2 | 2.309(3) | C35 | C40 | 1.391(12) |
| Pt2 | Cl3 | 2.286(3) | C23 | C24 | 1.425(13) |
| Pt2 | Cl1 | 2.290(4) | C23 | C28 | 1.365(13) |
| Ru1 | P3 | 2.375(2) | C41 | C42 | 1.403(11) |
| Ru1 | P1 | 2.309(2) | C41 | C46 | 1.386(12) |
| Ru1 | P2 | 2.391(2) | C48 | C47 | 1.420(13) |
| Ru1 | C48 | 2.220(9) | C48 | C49 | 1.418(13) |
| Ru1 | C47 | 2.210(9) | C47 | C51 | 1.382(13) |
| Ru1 | C50 | 2.228(8) | C50 | C49 | 1.402(13) |
| Ru1 | C49 | 2.243(8) | C50 | C51 | 1.394(13) |
| Ru1 | C51 | 2.219(8) | C18 | C19 | 1.389(13) |
| P3 | C29 | 1.837(8) | C36 | C37 | 1.395(13) |
| P3 | C35 | 1.834(8) | C42 | C43 | 1.365(13) |
| P3 | C41 | 1.841(8) | C33 | C34 | 1.390(12) |
| P1 | C1 | 1.828(9) | C33 | C32 | 1.371(13) |
| P1 | C2 | 1.848(9) | C22 | C21 | 1.391(14) |
| P1 | C3 | 1.834(9) | C46 | C45 | 1.398(13) |
| P2 | C11 | 1.835(9) | C12 | C13 | 1.374(13) |
| P2 | C17 | 1.841(9) | C16 | C15 | 1.378(14) |
| P2 | C23 | 1.844(9) | C40 | C39 | 1.407(13) |
| N2 | C3 | 1.504(10) | C32 | C31 | 1.376(14) |
| N2 | C5 | 1.503(11) | C37 | C38 | 1.342(15) |
| N2 | C7 | 1.482(12) | C13 | C14 | 1.366(15) |
| O1 | C8 | 1.235(13) | C38 | C39 | 1.367(15) |
| N4 | C10 | 1.530(14) | C24 | C25 | 1.362(13) |
| N4 | C8 | 1.235(14) | C45 | C44 | 1.370(14) |
| N4 | C9 | 1.491(15) | C30 | C31 | 1.406(13) |
| N1 | C1 | 1.503(11) | C14 | C15 | 1.381(15) |
| N1 | C4 | 1.531(14) | C43 | C44 | 1.390(14) |
| N1 | C6 | 1.537(12) | C20 | C21 | 1.383(15) |
| C29 | C34 | 1.380(12) | C20 | C19 | 1.351(15) |
| C29 | C30 | 1.390(11) | C28 | C27 | 1.402(15) |
| N3 | C2 | 1.468(11) | C25 | C26 | 1.382(17) |
| N3 | C5 | 1.399(12) | C27 | C26 | 1.350(18) |
| N3 | C4 | 1.435(13) |  |  |  |

| **Table S4**. Bond Angles for **2**. | | | | | | | |
| --- | --- | --- | --- | --- | --- | --- | --- |
| **Atom** | **Atom** | **Atom** | **Angle/˚** | **Atom** | **Atom** | **Atom** | **Angle/˚** |
| O1 | Pt1 | N2 | 93.5(3) | C34 | C29 | P3 | 118.3(6) |
| O1 | Pt1 | N1 | 177.3(3) | C34 | C29 | C30 | 117.5(8) |
| O1 | Pt1 | C10 | 82.6(4) | C30 | C29 | P3 | 124.2(6) |
| N1 | Pt1 | N2 | 87.3(3) | C5 | N3 | C2 | 114.6(8) |
| N1 | Pt1 | C10 | 96.6(4) | C5 | N3 | C4 | 116.8(9) |
| C10 | Pt1 | N2 | 175.8(4) | C4 | N3 | C2 | 114.8(8) |
| Cl4 | Pt2 | Cl2 | 178.96(13) | C12 | C11 | P2 | 121.5(7) |
| Cl4 | Pt2 | Cl1 | 88.78(15) | C12 | C11 | C16 | 117.6(8) |
| Cl3 | Pt2 | Cl4 | 91.50(14) | C16 | C11 | P2 | 120.8(7) |
| Cl3 | Pt2 | Cl2 | 88.45(14) | C18 | C17 | P2 | 121.9(7) |
| Cl3 | Pt2 | Cl1 | 179.11(14) | C22 | C17 | P2 | 120.0(7) |
| Cl1 | Pt2 | Cl2 | 91.26(15) | C22 | C17 | C18 | 118.1(8) |
| P3 | Ru1 | P2 | 100.79(7) | C36 | C35 | P3 | 123.1(7) |
| P1 | Ru1 | P3 | 94.74(8) | C36 | C35 | C40 | 117.9(8) |
| P1 | Ru1 | P2 | 98.21(8) | C40 | C35 | P3 | 119.0(7) |
| C48 | Ru1 | P3 | 109.4(3) | C24 | C23 | P2 | 120.1(7) |
| C48 | Ru1 | P1 | 92.8(3) | C28 | C23 | P2 | 123.2(8) |
| C48 | Ru1 | P2 | 146.9(3) | C28 | C23 | C24 | 116.6(9) |
| C48 | Ru1 | C50 | 61.4(3) | C42 | C41 | P3 | 120.9(6) |
| C48 | Ru1 | C49 | 37.1(3) | C46 | C41 | P3 | 121.0(6) |
| C47 | Ru1 | P3 | 89.0(3) | C46 | C41 | C42 | 117.4(8) |
| C47 | Ru1 | P1 | 126.5(2) | N1 | C1 | P1 | 117.5(6) |
| C47 | Ru1 | P2 | 133.4(2) | C47 | C48 | Ru1 | 70.9(5) |
| C47 | Ru1 | C48 | 37.4(3) | C49 | C48 | Ru1 | 72.3(5) |
| C47 | Ru1 | C50 | 60.8(3) | C49 | C48 | C47 | 106.7(9) |
| C47 | Ru1 | C49 | 61.5(3) | C48 | C47 | Ru1 | 71.7(5) |
| C47 | Ru1 | C51 | 36.4(3) | C51 | C47 | Ru1 | 72.2(5) |
| C50 | Ru1 | P3 | 141.5(2) | C51 | C47 | C48 | 109.0(9) |
| C50 | Ru1 | P1 | 121.9(2) | C49 | C50 | Ru1 | 72.3(5) |
| C50 | Ru1 | P2 | 86.5(2) | C51 | C50 | Ru1 | 71.4(5) |
| C50 | Ru1 | C49 | 36.5(3) | C51 | C50 | C49 | 109.2(8) |
| C49 | Ru1 | P3 | 146.3(3) | C19 | C18 | C17 | 120.0(9) |
| C49 | Ru1 | P1 | 91.0(2) | C35 | C36 | C37 | 121.0(9) |
| C49 | Ru1 | P2 | 111.2(3) | C43 | C42 | C41 | 122.1(9) |
| C51 | Ru1 | P3 | 105.1(2) | N3 | C2 | P1 | 107.5(6) |
| C51 | Ru1 | P1 | 151.7(2) | C32 | C33 | C34 | 119.1(9) |
| C51 | Ru1 | P2 | 97.7(2) | N2 | C3 | P1 | 115.8(6) |
| C51 | Ru1 | C48 | 61.8(3) | C21 | C22 | C17 | 120.8(10) |
| C51 | Ru1 | C50 | 36.5(3) | C29 | C34 | C33 | 122.3(8) |
| C51 | Ru1 | C49 | 61.4(3) | C41 | C46 | C45 | 120.7(9) |
| C29 | P3 | Ru1 | 115.5(3) | C13 | C12 | C11 | 120.2(9) |
| C29 | P3 | C41 | 99.0(4) | C48 | C49 | Ru1 | 70.6(5) |
| C35 | P3 | Ru1 | 120.1(3) | C50 | C49 | Ru1 | 71.1(5) |
| C35 | P3 | C29 | 104.7(4) | C50 | C49 | C48 | 107.3(8) |
| C35 | P3 | C41 | 98.9(4) | C15 | C16 | C11 | 120.8(9) |
| C41 | P3 | Ru1 | 115.5(3) | C35 | C40 | C39 | 120.8(9) |
| C1 | P1 | Ru1 | 119.1(3) | C47 | C51 | Ru1 | 71.5(5) |
| C1 | P1 | C2 | 97.3(4) | C47 | C51 | C50 | 107.9(8) |
| C1 | P1 | C3 | 106.5(4) | C50 | C51 | Ru1 | 72.1(5) |
| C2 | P1 | Ru1 | 116.7(3) | C33 | C32 | C31 | 120.8(9) |
| C3 | P1 | Ru1 | 118.4(3) | C38 | C37 | C36 | 119.9(11) |
| C3 | P1 | C2 | 94.3(4) | C14 | C13 | C12 | 122.3(10) |
| C11 | P2 | Ru1 | 114.7(3) | C37 | C38 | C39 | 121.7(10) |
| C11 | P2 | C17 | 99.6(4) | C25 | C24 | C23 | 122.0(10) |
| C11 | P2 | C23 | 101.4(4) | N3 | C5 | N2 | 113.1(7) |
| C17 | P2 | Ru1 | 111.0(3) | C44 | C45 | C46 | 120.5(10) |
| C17 | P2 | C23 | 102.8(4) | N3 | C4 | N1 | 113.7(8) |
| C23 | P2 | Ru1 | 124.1(3) | C29 | C30 | C31 | 121.0(9) |
| C3 | N2 | Pt1 | 108.7(5) | C13 | C14 | C15 | 118.8(10) |
| C5 | N2 | Pt1 | 113.3(6) | C42 | C43 | C44 | 119.7(9) |
| C5 | N2 | C3 | 109.3(7) | C19 | C20 | C21 | 120.4(10) |
| C7 | N2 | Pt1 | 110.8(6) | C20 | C21 | C22 | 119.5(10) |
| C7 | N2 | C3 | 107.0(7) | C23 | C28 | C27 | 120.9(11) |
| C7 | N2 | C5 | 107.5(8) | C38 | C39 | C40 | 118.8(10) |
| C8 | O1 | Pt1 | 113.2(8) | C16 | C15 | C14 | 120.2(10) |
| C8 | N4 | C10 | 122.7(10) | N4 | C10 | Pt1 | 100.6(7) |
| C8 | N4 | C9 | 122.4(11) | C45 | C44 | C43 | 119.6(10) |
| C9 | N4 | C10 | 114.8(10) | C32 | C31 | C30 | 119.3(9) |
| C1 | N1 | Pt1 | 107.8(6) | C20 | C19 | C18 | 121.0(10) |
| C1 | N1 | C4 | 109.0(7) | C24 | C25 | C26 | 119.5(12) |
| C1 | N1 | C6 | 106.6(8) | C26 | C27 | C28 | 121.0(12) |
| C4 | N1 | Pt1 | 114.2(6) | C27 | C26 | C25 | 119.8(11) |
| C4 | N1 | C6 | 104.7(8) | N4 | C8 | O1 | 120.8(12) |
| C6 | N1 | Pt1 | 114.2(7) |  |  |  |  |

**References**

(1) Daigle, D. J.; Pepperman, A. B.; Vail, S. L. Synthesis of a Monophosphorus Analog of Hexamethylenetetramine. *J Heterocycl Chem* **1974**, *11* (3), 407–408. https://doi.org/10.1002/JHET.5570110326.

(2) Mena-Cruz, A.; Lorenzo-Luis, P.; Romerosa, A.; Saoud, M.; Serrano-Ruiz, M. Synthesis of the Water Soluble Ligands DmPTA and DmoPTA and the Complex [RuClCp(HdmoPTA)(PPh3)](OSO2CF3) (DmPTA = N,N′-Dimethyl-1,3,5-Triaza-7-Phosphaadamantane, DmoPTA = 3,7-Dimethyl-1,3,7-Triaza-5-Phosphabicyclo[3.3.1]Nonane, HdmoPTA = 3,7-H-3,7-Dimethyl-1,3,7-Triaza-5-Phosphabicyclo[3.3.1]Nonane). *Inorg Chem* **2007**, *46* (15), 6120–6128. https://doi.org/10.1021/IC070168M/SUPPL_FILE/IC070168MSI20070130_104513.CIF.

(3) Gonzalez, B.; Lorenzo-Luis, P.; Romerosa, A.; Serrano-Ruiz, M.; Gili, P. Theoretical Aspects on Water Soluble [RuClCp(PPh3)2], [RuClCp(PTA)(PPh3)], [RuClCp(PTA)2], [RuClCp(MPTA)(PPh3)]+ and [RuClCp(MPTA)2]2+ (PTA = 1,3,5-Triaza-7-Phosphaadamantane; MPTA = N-Methyl-1,3,5-Triaza-7-Phosphaadamantane). *Journal of Molecular Structure: THEOCHEM* **2009**, *894* (1–3), 59–63. https://doi.org/10.1016/J.THEOCHEM.2008.09.042.

(4) Mendoza, Z.; Lorenzo-Luis, P.; Serrano-Ruiz, M.; Martín-Batista, E.; Padrón, J. M.; Scalambra, F.; Romerosa, A. Synthesis and Antiproliferative Activity of [RuCp(PPh3)2(HdmoPTA)](OSO2CF3)2 (HdmoPTA = 3,7-H-3,7-Dimethyl-1,3,7-Triaza-5-Phosphabicyclo[3.3.1]Nonane). *Inorg Chem* **2016**, *55* (16), 7820–7822. https://doi.org/10.1021/ACS.INORGCHEM.6B01207.

(5) Mendoza, Z.; Lorenzo-Luis, P.; Scalambra, F.; Padrón, J. M.; Romerosa, A. Enhancement of the Antiproliferative Activity of [RuCp(PPh3)2(DmoPTA-1κP)]+via Its Coordination to One {CoCl2} Unit: Synthesis, Crystal Structure and Properties of [RuCp(PPh3)2-μ-DmoPTA-1κP:2κ2N,N′-CoCl2](OTf)·0.25H2O. *Dalton Transactions* **2017**, *46* (25), 8009–8012. https://doi.org/10.1039/C7DT01741C.

(6) Mendoza, Z.; Lorenzo-Luis, P.; Scalambra, F.; Padrón, J. M.; Romerosa, A. One Step Up in Antiproliferative Activity: The Ru-Zn Complex [RuCp(PPh3)2-µ-DmoPTA-1κP:2κ2N,N′-ZnCl2](CF3SO3). *Eur J Inorg Chem* **2018**, *2018* (43), 4684–4688. https://doi.org/10.1002/EJIC.201800857.

(7) Sheldrick, G. M.; IUCr. SHELXT – Integrated Space-Group and Crystal-Structure Determination. *urn:issn:2053-2733* **2015**, *71* (1), 3–8. https://doi.org/10.1107/S2053273314026370.

(8) Sheldrick, G. M.; IUCr. Crystal Structure Refinement with SHELXL. *urn:issn:2053-2296* **2015**, *71* (1), 3–8. https://doi.org/10.1107/S2053229614024218.

(9) Dolomanov, O. V.; Bourhis, L. J.; Gildea, R. J.; Howard, J. A. K.; Puschmann, H. OLEX2: A Complete Structure Solution, Refinement and Analysis Program. *urn:issn:0021-8898* **2009**, *42* (2), 339–341. https://doi.org/10.1107/S0021889808042726.
